# Effectiveness of COVID-19 vaccines against SARS-CoV-2 variants of concern in real-world: a literature review and meta-analysis

**DOI:** 10.1080/22221751.2022.2122582

**Published:** 2022-09-29

**Authors:** Weihao Shao, Xiaorui Chen, Caifang Zheng, Haoshuang Liu, Gaili Wang, Bowen Zhang, Zhiyuan Li, Weidong Zhang

**Affiliations:** Department of Epidemiology, School of Public Health, Zhengzhou University, Zhengzhou, People’s Republic of China

**Keywords:** COVID-19 vaccine effectiveness, variants of concern, real-world study, meta-analysis, SARS-CoV-2

## Abstract

Knowing vaccine effectiveness (VE) against variants of concern (VOCs) in the real-world setting is essential for public health decision-making. A systematic landscape of the VE against a series of clinical outcomes caused by the VOCs in the real-world setting is needed. We systematically searched for studies that evaluated VE against VOCs in the real-world setting and collected individual data. We identified 113 studies meeting the eligibility criteria. We found full vaccination provided strong protection against each clinical outcome with summary VE ranging from 86.8% to 96.0% Alpha, moderate protection against infection caused by Beta, Gamma and Delta with summary VE ranging from 70.9% to 72.8%, strong protection against severe disease caused by Delta with summary VE ranging from 84.9% to 90.3%, limited protection with summary VE of 23.5% (95% CI, 17.0–29.5) against infection and moderate protection with summary VE ranging from 56.5% to 82.4% against severe diseases caused by Omicron. Booster vaccination can provide a substantial improvement in protection against Delta and Omicron, but not as much as the Delta. The meta-regression analysis showed that the VE against the Omicron wanned over time, and the VE against hospitalization declined relatively slowly, compared to against infection. Those findings supported the need for public health measures, increasing booster vaccination coverage in response to current and new infectious waves driven by variants and developing broadly protective vaccines to confront virus evolution.

## Introduction

The coronavirus disease 2019 (COVID-19) pandemic has had a significant impact worldwide, with over 584 million cases and 6.41 million COVID-19-related deaths reported globally as of 11 August 2022 [1]. More serious infections and transmission have been exacerbated by the emergence of variants worldwide, in particular the recent emergence of the Omicron variant, which has given rise to an unprecedented wave of infections [2].

The COVID-19 vaccination campaign is the most essential component of the current response to the pandemic, helping to reduce the COVID-19 disease burden, facilitate a safer reopening of societies and a recovery of the economy. As of 4 June 2022, 32 COVID-19 vaccines have been approved for use and 129 are in clinical trials [3]. Mass vaccination campaigns are under way in many countries, and booster vaccination schedules are being implemented. The immunogenicity, safety and efficacy of many vaccines are well supported by randomized controlled trials (RCT) or observational studies [4–6]. However, most COVID-19 vaccines have been developed for early pandemic strains, and the emergence of new variants of concern (VOCs) including Alpha, Beta, Gamma, Delta and Omicron has challenged the vaccine effectiveness (VE) [7]. Multiple studies have reported that the infectivity and immune evasion ability of the VOCs, as compared with original strains, are increased and the VE against the VOCs was lower and wanning over time swiftly. To the best of our knowledge, there have been some RCT studies reporting the VE against various clinical outcomes caused by the VOCs [8–11]. However, RCT studies are conducted in a very demanding study setting, with strict restrictions on the study population and trial conditions. The study setting was significantly different from the real-world study. The performance of VE in the real-world setting is affected by public health measures, individual self-protective behaviours, access to health services, vaccine hesitancy and a wide range of heterogeneous study populations [12,13].

The performance of COVID-19 vaccines in the real-world setting is a critical assessment of evidence-based public health decisions. Although some studies have reported the real-world effectiveness of COVID-19 vaccines against VOCs, the results reported by various studies are controversial [14–17]. Meta-analysis based on real-world evidence is urgently needed for comprehensive evaluation. In this study, we aimed to systematically evaluate the VE in the real-world setting against each clinical outcome caused by the VOCs.

## Methods

This systematic review and meta-analysis were reported in accordance with the Preferred Reporting Items for Systematic Reviews and Meta-Analyses (PRISMA) guidelines [18]. The protocol for this study was registered and accepted in the International Prospective Register of PROSPERO (CRD42022334369).

### Search strategy

Using the keywords “COVID-19,” “SARS-CoV-2,” “vaccine,” and “variant” (supplementary table S3 for search strategy), we systematic searched for literature published on PubMed, Cochrane Library, Embase before 5th Aug 2022. To ensure the validity of study results, studies published on preprint servers without peer review were not retrieved and included. Additionally, we reviewed the references from the included studies to identify any missed potentially relevant records.

### Selection of studies and data extraction

Observational studies (cohort, case–control, test-negative case–control) that evaluated the VE against VOCs including B.1.1.7 (Alpha), B.1.351 (Beta), P.1 (Gamma), B.1.617.2 (Delta) and B.1.152.9 (Omicron) were included. Clinical trials, studies on immunogenicity and antibody neutralization, estimation of comparative or marginal effectiveness and hybrid immunity (the protection against VOCs conferred by natural immunity and vaccination), studies on non-VOCs, case reports, conference abstracts, cross-sectional and ecological studies and mathematical modelling analysis studies were excluded. The adjusted VE or estimates of effect size (odds ratio, relative risk, hazard ratio and incidence rate ratio) in various vaccination statuses (partial, full and booster) against a series of clinical outcomes caused by VOCs with corresponding 95% confidence intervals (CIs) was extracted preferentially. We also extracted the first authors’ name, VE at each time interval, publication year, specific VOCs type, clinical outcomes reported in each article, study design, country and sample size according to a predetermined proforma in Microsoft Excel spreadsheet.

### Risk of bias assessment

The methodological quality of each study was assessed independently by two authors using the original Newcastle-Ottawa Scale (NOS) for case–control and cohort studies [19]. The NOS contains 8 items with scores ranging from 0 to 9 points. The total score of 0–3, 4–6, and 7–9 points indicated low, moderate and high quality, respectively. Any discrepancies were resolved by discussion until unanimous consensus was reached.

### Statistical analysis

We estimated the summary VE against a range of clinical outcomes caused by VOCs in line with the vaccination status (vaccination status varied according to the recommendation of the local health department in each study population). VE was obtained from the effect size (odds ratio, relative risk, hazard ratio and incidence rate ratio) defining as (1-effect size) ×100%. Summary estimates and 95% CIs were calculated using DerSimonian and Laird random-effects meta-analysis. We used the meta-regression based on a linear mixed model to estimate the change of VE over time to focus on the most concerned Omicron variant. Predetermined subgroup analysis was performed stratifying by vaccine product and study population to explore the VE in specific populations and the performance of various vaccine products. Heterogeneity between included studies was assessed by the I-square statistics using the following interpretation: 0–50%, 50–75% and >75% were considered to be low, moderate and high heterogeneity respectively. The forest plot was used to present the results of summary VE and subgroup analyses against each clinical outcome caused by VOCs and the corresponding study heterogeneity. To ensure the robustness of the results, we did not perform summary estimation for clinical outcome or subgroup with less than three studies. Publication bias was assessed by visual inspection for funnel plot asymmetry and by the Egger’s test. When publication bias was examined based on either Egger’s test of bias or visual inspection for funnel plot asymmetry, we used the trim-and-fill method to re-estimate the pooled effect size. Additionally, the sensitivity analyses of all outcomes were performed to assess the influence of included study on the results. All calculations and graphs were conducted using R software version 4.1.2 (R Foundation for Statistical Computing).

## Results

### Characteristics of the included studies

**Figure 1.**
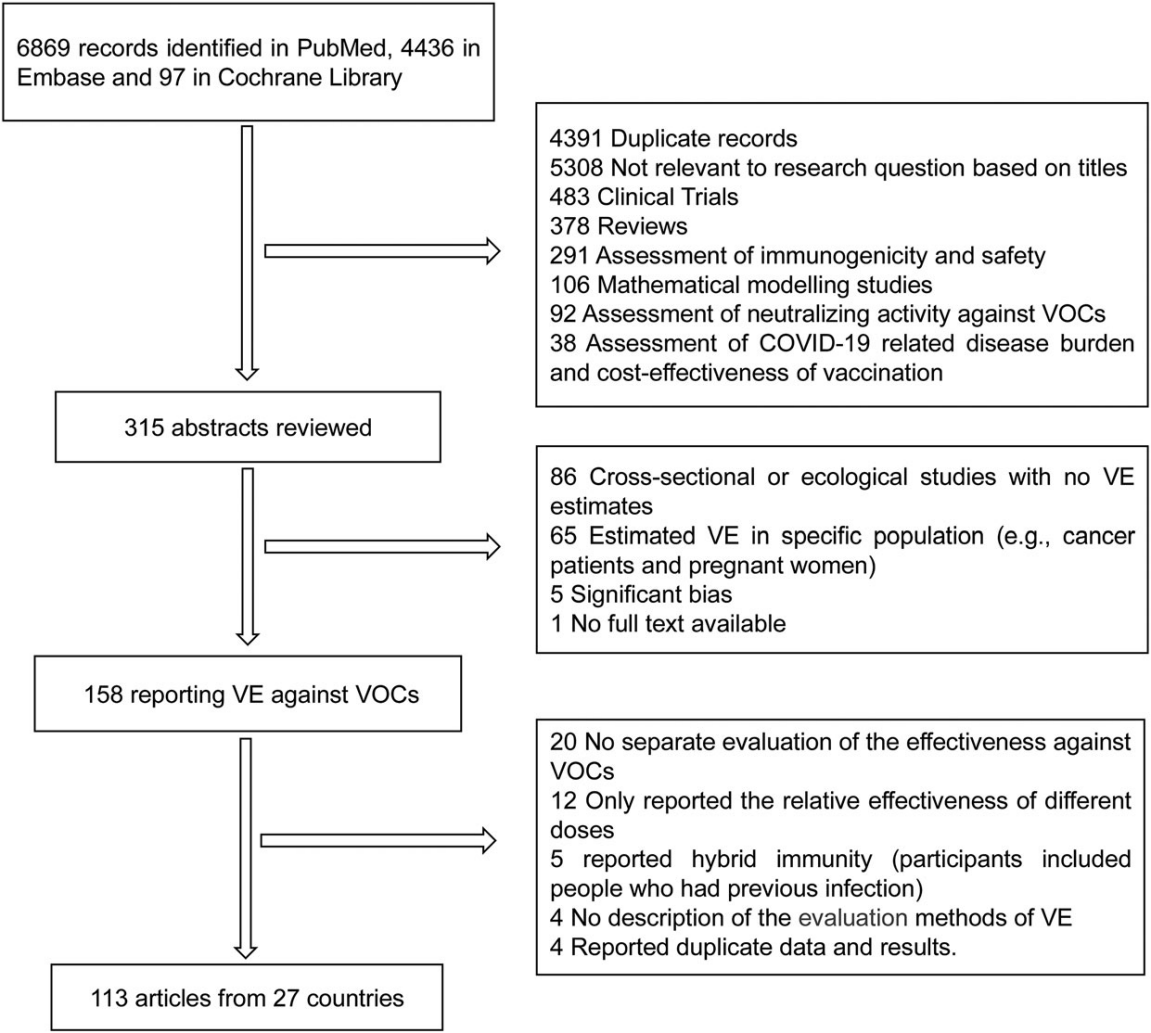
Flowchart of study selection

For this literature review, we identified 11,402 potentially relevant records from the literature databases, 4391 were excluded as duplicates and 5308 irrelevant studies based on title and abstract were excluded, 315 published articles were evaluated at the full-text level. In total, 113 articles including 51 test-negative case–control studies, 51 cohort studies, and 11 case–control studies were included from 27 countries (Figure 1). Of the 113 included studies, 8 COVID-19 vaccines (mRNA-1273, BNT162b2, ChAdOx1, Ad26.COV2.S, BBV152, CoronaVac, BBIBP-CorV and Gam-COVID-Vac) and 6 clinical outcomes including SARS-Cov-2 infection confirmed by PCR or antigen test, COVID-19 related hospitalization, COVID-19 associated emergency department or urgent care (ED or UC) visits, severe, critical or fatal COVID-19 disease, COVID-19 related intensive care unit (ICU) admission and COVID-19 related death (the interpretation of clinical outcomes of interest were shown in supplementary table S4) were included in this study. Among the 113 studies assessed using the NOS scale, 6 studies at moderate quality and 107 at high quality (supplementary table S2 for characteristics of included studies).

### Vaccine effectiveness of COVID-19 Vaccines against Alpha, Beta and Gamma variant

**Figure 2.**
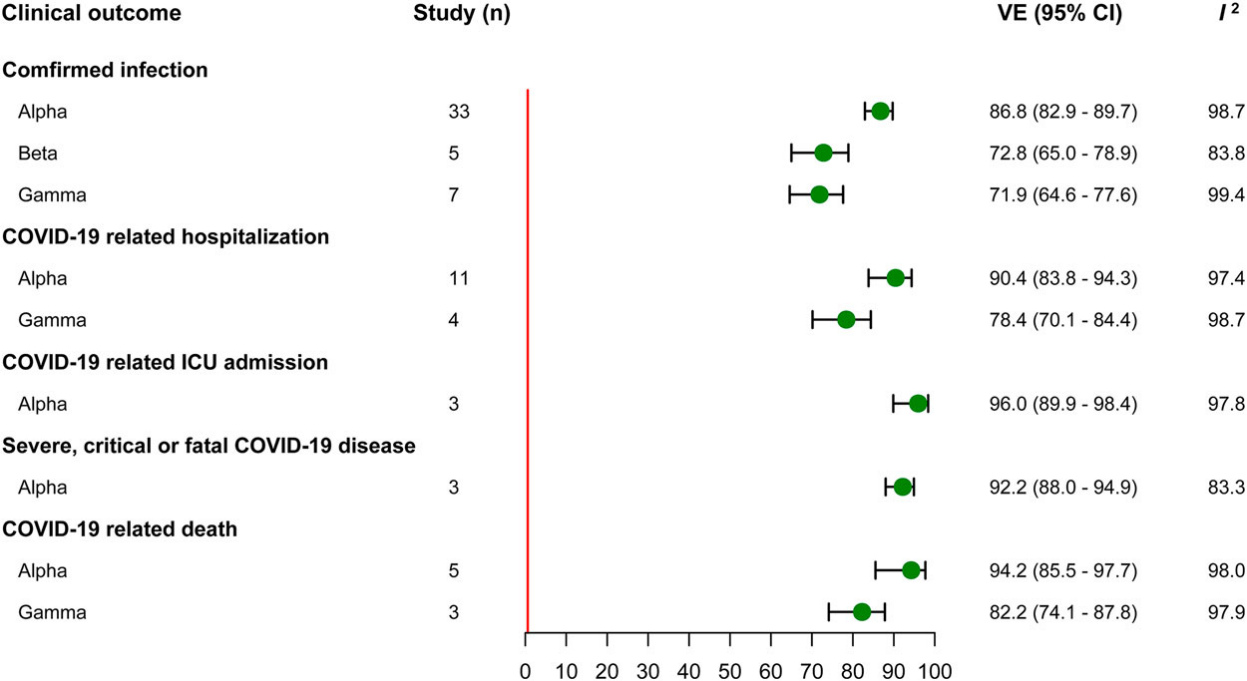
VE of full vaccination against Alpha, Beta and Gamma variant.

Thirty-three, five and seven studies evaluated the VE of full vaccination against infection caused by Alpha, Beta and Gamma variant, with a summary VE of 86.8% (95% CI, 82.9–89.7), 72.8% (95% CI, 65.0–78.9) and 71.9% (95% CI, 64.6–77.6) respectively. In the analyses of summary VE estimation against hospitalization, the summary VE was 90.4% (95% CI, 83.8–94.3) for Alpha variant and 78.4% (95% CI, 70.1–84.4) for Gamma variant. Owing to limited evidence, only three studies evaluated the VE against ICU admission and severe disease caused by Alpha variant, and the summary VE was 96.0% (95% CI, 89.9–98.4) and 92.2% (95% CI, 88.0–94.9) respectively. Five and three studies provided VE estimation of full vaccination against death caused by Alpha and Gamma variant, giving a summary VE of 94.2% (95% CI, 85.5–97.7) and 82.2% (95% CI, 74.1–87.8) (Figure 2). In addition, Subgroup analysis of full vaccination by vaccine products and study population and VE estimation of partial vaccination were also conducted, and the results were presented in the supplementary table S4-S6.

### Vaccine effectiveness of COVID-19 vaccines against B.1.617.2 (Delta) variant

**Figure 3.**
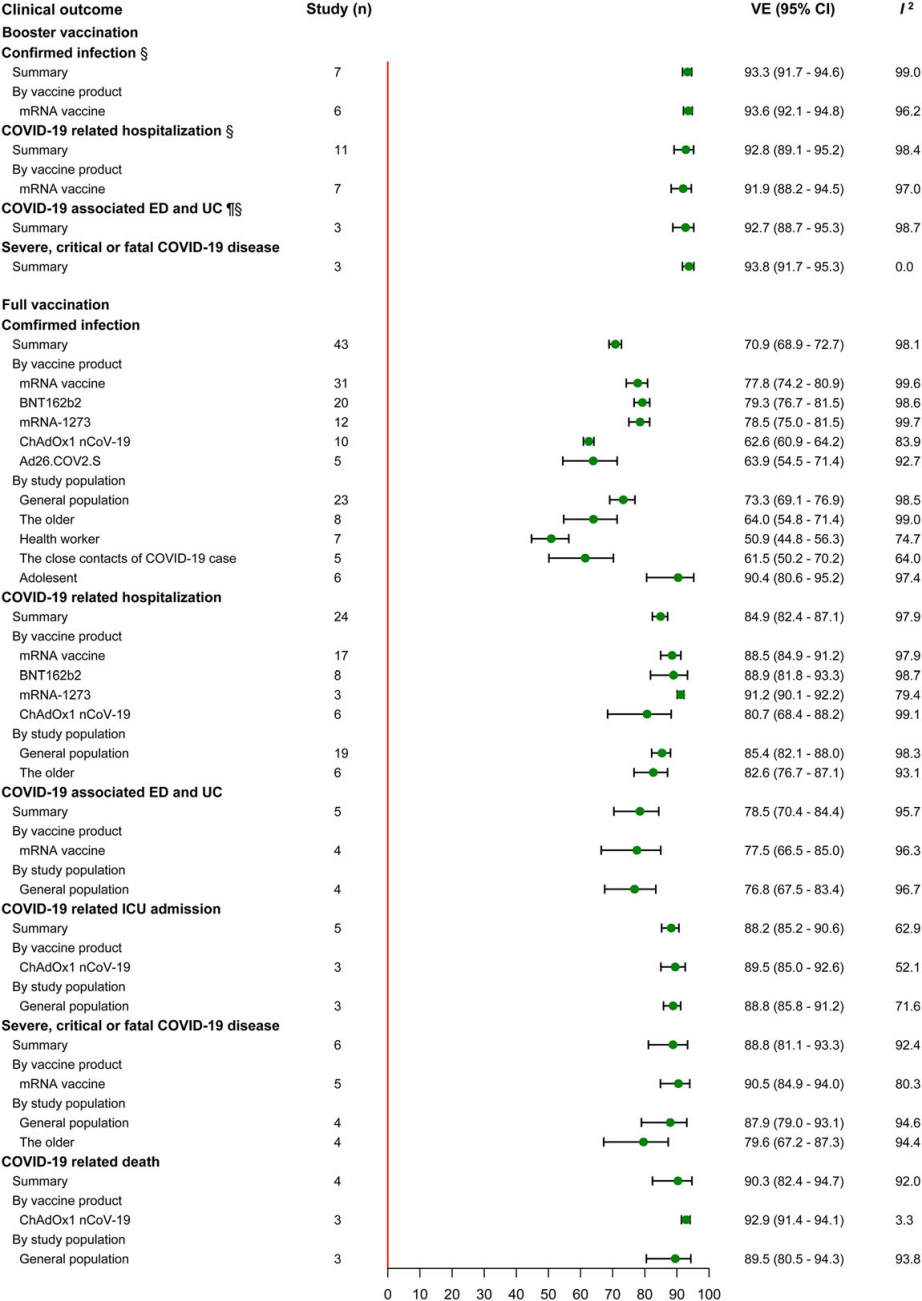
VE of booster and full vaccination against Delta variant. Studies that estimated VE all used mRNA vaccines. ^§^All studies conducted in general population.

Seven, eleven and three studies provided VE estimation of booster vaccination against infection, hospitalization and ED or UC caused by Delta variant among the general population, with summary VE of 93.3% (95% CI, 91.7–94.6), 92.8% (95% CI, 89.1–95.2) and 92.7% (95% CI, 88.7–95.3) respectively. The VE against severe disease was evaluated in 3 studies, 2 among the general population and 1 among the elderly, giving a summary VE of 93.8% (95% CI, 91.7–95.3). The summary VE of full vaccination was 70.9% (95% CI, 68.9–72.7, 43 studies) against infection, 84.9% (95% CI, 82.4–87.1, 24 studies) against hospitalization, 78.5% (95% CI, 70.4–84.4, 5 studies) against ED or UC, 88.2% (95% CI, 85.2–90.6, 5 studies) against ICU admission, 88.8% (95% CI, 81.1–93.3, 6 studies) against severe disease and 90.3% (95% CI, 82.4–94.7, 4 studies) against death (Figure 3, Supplementary Table S7). Subgroup analyses by study population showed low summary VE against infection in high-risk populations, 64.0 (95% CI, 54.8–71.4, 8 studies) for the elderly, 51.1 (95% CI, 38.3–61.2, 6 studies) for HCW and 61.5 (95% CI, 50.2–70.2, 5 studies) for the close contacts of COVID-19 case. In contrast, the VE performed better among the general population and adolescent compared to the high-risk population with a summary VE of 73.3 (95% CI, 69.1–76.9, 23 studies) and 90.4 (95% CI, 80.6–95.2, 6 studies). In subgroup analyses of other clinical outcomes, subgroup VE by study population was also found to be lower among the elderly compared to general population and the ungrouped summary VE. In the subgroup analysis of vaccine product, we estimated the booster and full vaccinated VE of the two mRNA vaccines, ChAdOx1 nCoV-19 and Ad26.COV2.S. The mRNA vaccines appeared to perform better compared to ungrouped summary VE and adenovirus vaccine. Additionally, we also estimated VE of partial vaccination against each clinical outcome (Supplementary Table S7).

### Vaccine effectiveness of COVID-19 vaccines against B.1.1.529 (Omicron) variant

**Figure 4.**
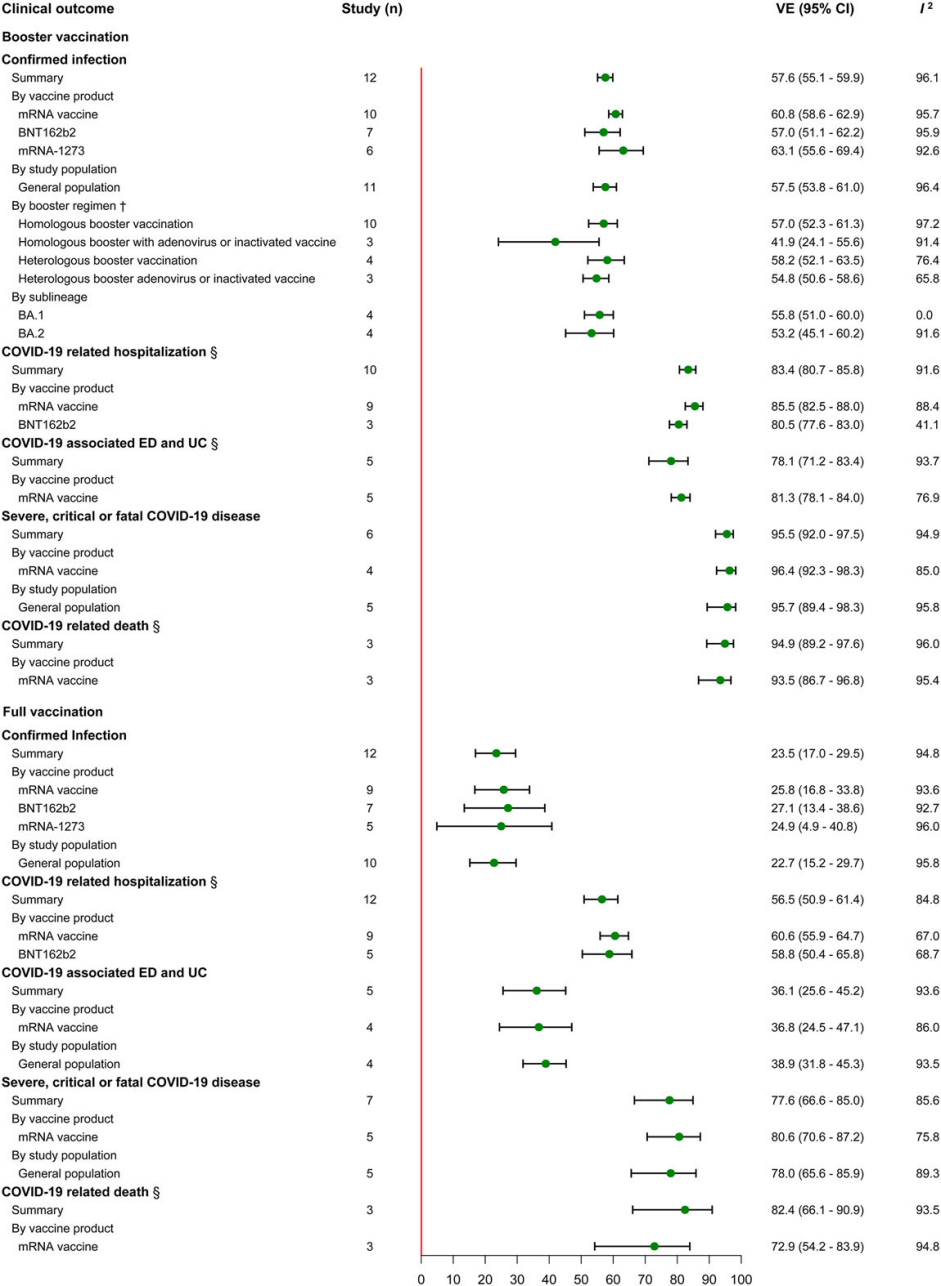
VE of booster and full vaccination against Omicron variant. ^†^Five COVID-19 vaccines were combined into six booster regimens including two dose ChAdOx1 nCoV-19 with BNT162b2 or mRNA-1273 booster, two dose BNT162b2 with mRNA-1273 booster, two dose mRNA-1273 with BNT162b2 booster, one dose Ad26.COV2-S with BNT162b2 or mRNA-1273 booster and two dose CoronaVac with BNT162b2 booster.

In studies of VE against Omicron variant, we estimated that the summary VE of booster vaccination was 57.6 (95% CI, 55.1–59.9, 12 studies) against infection, 83.4 (95% CI, 80.7–85.8, 10 studies) against hospitalization, 78.1 (95% CI, 71.2–83.4, 5 studies) against ED and UC, 95.5 (95% CI, 92.0–97.5, 6 studies) against severe disease and 94.9 (95% CI, 89.2–97.6, 3 studies) against death (Figure 4). Subgroup estimates by booster regimen showed that the summary VE was 57.0 (95% CI, 52.3–61.3, 10 studies) and 54.8 (95% CI, 50.6–58.6, 4 studies) for homologous and heterologous boosting. Particularly, we also compared the summary VE against infection of homologous and heterologous boosting of adenovirus and inactivated vaccine, with summary VE of 41.9 (95% CI, 24.1–55.6, 3 studies) and 54.8 (95% CI, 50.6–58.6, 3 studies) respectively. The VE of Omicron sublineage was estimated in four studies, giving a summary VE of 55.8 (95% CI, 51.0–60.0) for BA.1 and 53.2 (95% CI, 45.1–60.2) for BA.2. Our summary estimated showed the low VE of full vaccination against Omicron variant, 23.5% (95% CI, 17.0–29.5, 12 studies) against infection, 56.5% (95% CI, 50.9–61.4, 12 studies) against hospitalization, 36.1% (95% CI, 25.6–45.2, 5 studies) against ED and UC, 77.6% (95% CI, 66.6–85.0, 7 studies) against severe disease and 82.4% (95% CI, 66.1–90.9, 3 studies) against death. Subgroup estimates by vaccine product revealed that the summary VE of mRNA vaccines was relatively higher against infection at 60.8% (95% CI, 58.6–62.9, 10 studies), against hospitalization at 85.5% (95% CI, 82.5–88.0, 9 studies) and against ED or UC at 81.3% (95% CI, 78.1–84.0, 5 studies) compared to the ungrouped summary VE. Similarly, a consistent pattern was observed in the analysis of full vaccination. Additionally, we also estimated the summary VE of partial vaccination against infection, with a summary VE of 25.9% (95% CI, 20.0–34.9) estimated in 5 studies (Supplementary Table S9).

### Vaccine effectiveness against Omicron variants over time since vaccination

**Figure 5.**
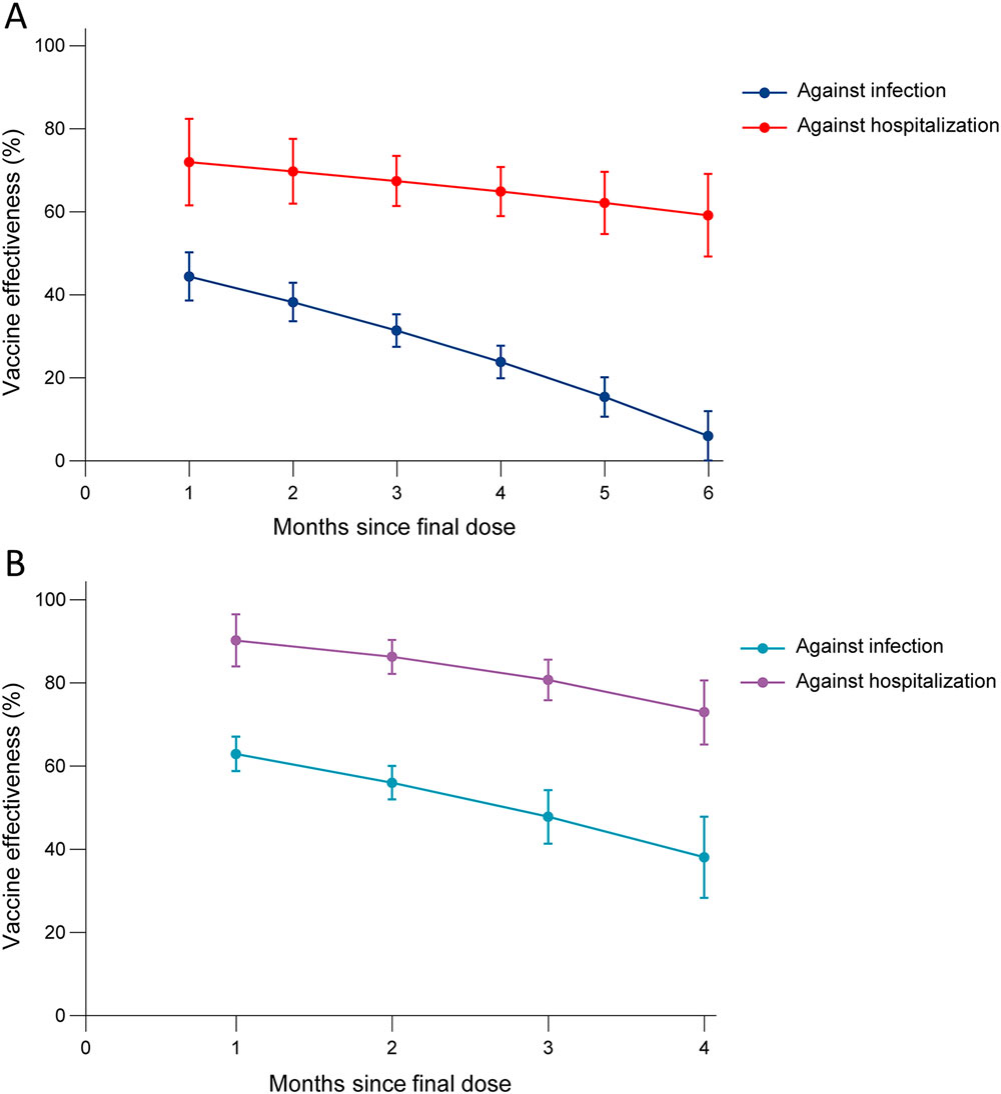
VE of (A) booster and (B) full vaccination against Omicron variant by time since vaccination.

Twelve studies evaluated the VE over time against infection and hospitalization caused by Omicron variant, among which were 5 vaccines (mRNA-1273, BNT162b2, ChAdOx1, Ad26.COV2.S and CoronaVac). The summary VE of full vaccination against infection was 44.4% (95% CI 38.6–50.2) at first month and subsequently declined, with VE of 6.0% (95% CI, 0.1–11.9) at sixth month. In comparison, the summary VE against hospitalization was 71.9% (95% CI, 61.5–82.4) at first month and declined slowly to 59.2% (95% CI, 49.2–69.2) at sixth month. Booster vaccination showed a relatively high protective effect but also wanning, declining from 62.9% (95% CI, 58.8–67.1) at first month to 38% (95% CI, 28.3–47.8) at fourth month. Compared with this, the summary VE against hospitalization still remained higher, wanning from 90.2% (95% CI, 83.9–96.5) at first month to 72.9% (95% CI, 65.2-80.6) at fourth month (Figure 5).

### Publication bias and sensitivity analysis

Publication bias was found with Egger’s test (*t *= −2.97, *P* value = 0.0054) in the analysis of VE for partial vaccination against infection caused by Alpha variant and we corrected the results using trim-and-fill method. No significant publication bias was identified in the remaining primary analyses, either qualitatively based on funnel-plot or visually based on funnel-plot. The sensitive analysis revealed that the results of primary and subgroup analyses were stable.

## Discussion

In this comprehensive systematic review and meta-analysis assessing the effectiveness COVID-19 vaccines against VOCs based on real-world studies, we found that full vaccination showed high VE against each clinical outcome caused by Alpha variant, moderate VE against infection caused by Beta, Gamma and Delta variant, high VE against severe disease caused by Delta variant, low VE against infection and moderate VE against severe disease caused by Omicron variant. Booster vaccination substantially increased VE against Delta and Omicron variants, with strong protection against each clinical outcome caused by Delta variant but with limited protection against infection and moderate protection against severe disease caused by Omicron.

Our results highlight the need for full and booster vaccination, especially in the unprecedented wave of infections caused by the Omicron variant. A national cohort study in Scotland showed a 56% (95% CI, 51–60) reduction in the odds of developing symptomatic infection 2 weeks or more after homologous or heterologous booster vaccination compared to 25 weeks or more after a primary series during the dominance of the Omicron variant [20]. The study conducted in Qatar demonstrated that booster vaccination with BNT162b2, as compared with that of the two-dose primary series, was associated with an 86.1% (95% CI, 67.3–94.1) reduction in the risk of symptomatic Delta infection, a 49.4% (95% CI, 47.1–51.6) reduction in the risk of symptomatic Omicron infection and a 76.5% (95% CI, 55.9–87.5) reduction in the risk of severe disease due to omicron variant [15]. Consistently, our results reveal that booster vaccination, although less effective against infection due to omicron variant, are essential in preventing severe disease.

Meta analysis of neutralizing antibody responses showed results that are generally consistent with the real-world study. Antibody in vaccine-induced serum was largely retained neutralizing response against Alpha variant. Nevertheless, the neutralizing response against Beta, Delta and Gamma variants was significantly reduced compared with wild type [21]. The assessment of neutralizing response showed Omicron variant, as compared with the wild type, significantly escaped neutralization induced by two doses of inactivated vaccines, with an 11.65-fold reduction.

At present, the booster vaccination is being carried out globally, and several studies indicated that booster vaccination can effectively induce B cells and strengthen T cell response to recognize VOCs [22-24]. Furthermore, the heterologous boost strategy has been initiated in many regions, particularly where inactivated or adenovirus vectors vaccine were used as the primary course to improve the VE. Our study shows that heterologous regimens present better VE against infection caused by the Omicron variant. The study for immunogenicity has shown that people receiving an mRNA booster after two doses of CoronaVac and ChAdOx1 nCoV-19 induced higher neutralizing antibody and better T cells response compared with homologous vaccination [25,26]. A network meta-analysis including 13 vaccine regimens also supports this finding [27].

Vaccine-induced neutralizing antibody titres appear to be a powerful predictor of VE against VOC. Studies based on in vitro neutralization assays showed that neutralizing antibody titres required to prevent severe disease were sixfold lower than symptomatic infections [28,29], which may explain why the VE in our findings with full and booster vaccination against severe disease caused by VOCs remain high. Our study showed that the VE of booster and full vaccination against Omicron wanned over time, especially against infection. One study showed that neutralizing activity rapidly decreases over time after vaccination, while T-cell responses are largely preserved, and the wanning for VE may be more related to humoral immunity [23]. The emerging Omicron sublineages that may evade neutralization and the decline of VE over time pose a challenge to vaccine efficacy [30], although we did not observe a significant difference in protection against infection with BA.1 and BA.2. In view of the current wave of Omicron variant infections and future wave driven by new variants, booster vaccination campaigns and the development of the next generation of vaccines target a broad range of coronaviruses to confront virus evolution appear to be priorities.

Another finding in our study is that the performance of vaccines in high-risk populations was less effective than that among the general population. Studies of immunogenicity assessment reveal that age differences can affect vaccine efficacy, with lower geometric mean titres (GMT) among the elder group compared to the young group, suggesting that the young group had better immunogenicity [31]. A study in the UK shows that the wanning of the VE against symptomatic diseases appeared to occur more frequently in the clinically vulnerable elder adults [32]. Consequently, the elderly may be preferentially recommended for vaccination in the booster vaccination campaign. Healthcare workers and the close contacts of SARS-Cov-2 positive cases have a higher frequency of antigen testing and a higher risk of exposure than the general population, which could explain the poor performance of vaccines among these high-risk populations. Our findings highlight the urgency of increasing vaccination coverage among these populations.

In addition, we also found that the VE of mRNA vaccine was relatively higher than that of other vaccine products. The evidence from clinical trials further supported our findings that mRNA vaccines are found to be better immunogenicity, with higher GMT and stronger cell responses compared to viral vector platforms [33,34]. The consistent pattern was observed in booster immunization, with an estimated 1.6-fold higher neutralizing activity and better variant-specific T-cell response for comparisons between homologous mRNA and inactivated or adenovirus vector platforms vaccine [24,35]. Additionally, the network meta-analysis showed that the BNT162b2 and mRNA-1273 vaccines had the highest probability of the best rank of efficacy against symptomatic diseases [36]. The immunogenicity and efficacy of COVID-19 vaccine are affected by many factors, including the principle of technology platform, vaccination regimen and population, etc. The effectiveness, safety, long-term efficacy and cost-effectiveness of different vaccine products should be taken into consideration when determining the priority of vaccine use.

This study has several limitations. Firstly, studies evaluating each VOC are out of proportion, and we did not evaluate VE against certain clinical outcomes caused by VOCs limited by the available evidence. For example, only six studies covered the Beta variant, and we only evaluated the effectiveness of the vaccine against infection. This was also used for subgroup analyses by study population and vaccine product. Second, the identification of vaccination status showed subtle differences in the studies we included. Most studies defined full vaccination as receipt of the second dose of COVID-19 vaccines (the first dose of Ad26.COV2.S ≥ 14 days) before the testing or other clinical outcomes occur. However, there were still a few studies that define 7 days or more instead of 14 days as full vaccination. The previous study reveals that this slight difference in follow-up time does not differ in evaluation of VE [37]. Initially, the third booster dose were offered 6 months after receipt of the primary course. The department of health of several countries shortened the interval from 6 months to 5 months or less in response to infection spikes due to Delta and Omicron variant [38,39], we did not differentiate this because there were no subgroup analyses by vaccination interval in the original study. Thirdly, in the meta-regression analysis to evaluate the wanning of VE over time, we only evaluated the wanning pattern in the general population due to insufficient studies. In addition, evidence for clinical trials of vaccines based on other platforms including protein subunits, DNA vaccines and live-attenuated vaccines have been published [40–42], but real-world evidence are still limited. Therefore, we did not evaluate vaccines based on these platforms in our analysis, and we can further explore their performances in the real world in the future.

In conclusion, our findings indicate that full vaccination provided strong protection against Alpha, moderate protection against infection caused by Beta, Gamma and Delta, strong protection against severe disease caused by Delta, limited protection against infection and moderate protection against severe diseases caused by Omicron. Booster vaccination can provide a substantial improvement in protection against Delta and Omicron, but not as much as the Delta. Meta-regression analysis showed that the VE against the Omicron wanned over time, and the VE against hospitalization declined relatively slowly compared to against infection. In the context of a global expansion of the omicron variant and infectious wave driven by new variants in the future, those finding supported the need for public health measures, increasing booster vaccination coverage and developing vaccines against a wide range of variants to confront virus evolution.

## Supplementary Material

Supplemental Material

## Data Availability

All data included were derived from publicly available documents cited in the references in the supplementary materials. Extracted data are available upon request to the corresponding author.
