# Supplementary material for: Effectiveness of COVID-19 vaccines against SARS-CoV-2 variants of concern in real-world: a literature review and meta-analysis
Source: Emerg Microbes Infect. 2022 Sep 29;11(1):2383–92. doi: 10.1080/22221751.2022.2122582 (PMC9542696; doi:10.1080/22221751.2022.2122582)
Supplement: Supplemental Material [file TEMI_A_2122582_SM5883.docx]

Supplementary Material

Table S1. PRISMA Checklist

Table S2. Characteristics of included studies in this systematic review and meta-analysis

Table S3. Search strategy for peer-reviewed databases.

Table S4. Interpretation of clinical outcomes of interest.

Table S5. VE of full and partial vaccination against Alpha variant.

Table S6. VE of full and partial vaccination against Beta variant.

Table S7. VE of full and partial vaccination against Gamma variant.

Table S8. VE of booster, full and partial vaccination against Delta variant.

Table S9. VE of booster, full and partial vaccination against Omicron variant.

Table S1. PRISMA Checklist

| **Section and Topic** | **Item #** | **Checklist item** | **Location where item is reported** |
| --- | --- | --- | --- |
| **TITLE** | | |  |
| Title | 1 | Identify the report as a systematic review. | Page 1 |
| **ABSTRACT** | | |  |
| Abstract | 2 | See the PRISMA 2020 for Abstracts checklist. | Page 2 |
| **INTRODUCTION** | | |  |
| Rationale | 3 | Describe the rationale for the review in the context of existing knowledge. | Page 3-4 |
| Objectives | 4 | Provide an explicit statement of the objective(s) or question(s) the review addresses. | Page 4 |
| **METHODS** | | |  |
| Eligibility criteria | 5 | Specify the inclusion and exclusion criteria for the review and how studies were grouped for the syntheses. | Page 5, Figure 1 |
| Information sources | 6 | Specify all databases, registers, websites, organisations, reference lists and other sources searched or consulted to identify studies. Specify the date when each source was last searched or consulted. | Page 5, Figure 1, Supplementary table S3 |
| Search strategy | 7 | Present the full search strategies for all databases, registers and websites, including any filters and limits used. | Page 5, Supplementary table S3 |
| Selection process | 8 | Specify the methods used to decide whether a study met the inclusion criteria of the review, including how many reviewers screened each record and each report retrieved, whether they worked independently, and if applicable, details of automation tools used in the process. | Figure 1 |
| Data collection process | 9 | Specify the methods used to collect data from reports, including how many reviewers collected data from each report, whether they worked independently, any processes for obtaining or confirming data from study investigators, and if applicable, details of automation tools used in the process. | Page 5-6 |
| Data items | 10a | List and define all outcomes for which data were sought. Specify whether all results that were compatible with each outcome domain in each study were sought (e.g. for all measures, time points, analyses), and if not, the methods used to decide which results to collect. | Page 6, Page 8 |
|  | 10b | List and define all other variables for which data were sought (e.g. participant and intervention characteristics, funding sources). Describe any assumptions made about any missing or unclear information. | Page 6, Supplementary table S2 |
| Study risk of bias assessment | 11 | Specify the methods used to assess risk of bias in the included studies, including details of the tool(s) used, how many reviewers assessed each study and whether they worked independently, and if applicable, details of automation tools used in the process. | Page 6 |
| Effect measures | 12 | Specify for each outcome the effect measure(s) (e.g. risk ratio, mean difference) used in the synthesis or presentation of results. | Page 6 |
| Synthesis methods | 13a | Describe the processes used to decide which studies were eligible for each synthesis (e.g. tabulating the study intervention characteristics and comparing against the planned groups for each synthesis (item #5)). | Page 5, Page 8, Figure 1 |
|  | 13b | Describe any methods required to prepare the data for presentation or synthesis, such as handling of missing summary statistics, or data conversions. | Page 6-7 |
|  | 13c | Describe any methods used to tabulate or visually display results of individual studies and syntheses. | Page 8 |
|  | 13d | Describe any methods used to synthesize results and provide a rationale for the choice(s). If meta-analysis was performed, describe the model(s), method(s) to identify the presence and extent of statistical heterogeneity, and software package(s) used. | Page 7-8 |
|  | 13e | Describe any methods used to explore possible causes of heterogeneity among study results (e.g. subgroup analysis, meta-regression). | Page 8 |
|  | 13f | Describe any sensitivity analyses conducted to assess robustness of the synthesized results. | Page 8 |
| Reporting bias assessment | 14 | Describe any methods used to assess risk of bias due to missing results in a synthesis (arising from reporting biases). | Page 8 |
| Certainty assessment | 15 | Describe any methods used to assess certainty (or confidence) in the body of evidence for an outcome. | Not applicable |
| **RESULTS** | | |  |
| Study selection | 16a | Describe the results of the search and selection process, from the number of records identified in the search to the number of studies included in the review, ideally using a flow diagram. | Page 9, Figure 1 |
|  | 16b | Cite studies that might appear to meet the inclusion criteria, but which were excluded, and explain why they were excluded. | Page 6, Figure 1 |
| Study characteristics | 17 | Cite each included study and present its characteristics. | Supplementary Table S2 |
| Risk of bias in studies | 18 | Present assessments of risk of bias for each included study. | Supplementary Table S2 |
| Results of individual studies | 19 | For all outcomes, present, for each study: (a) summary statistics for each group (where appropriate) and (b) an effect estimate and its precision (e.g. confidence/credible interval), ideally using structured tables or plots. | Supplementary Table S2 |
| Results of syntheses | 20a | For each synthesis, briefly summarise the characteristics and risk of bias among contributing studies. | Figure 2-5 |
|  | 20b | Present results of all statistical syntheses conducted. If meta-analysis was done, present for each the summary estimate and its precision (e.g. confidence/credible interval) and measures of statistical heterogeneity. If comparing groups, describe the direction of the effect. | Figure 2-5 |
|  | 20c | Present results of all investigations of possible causes of heterogeneity among study results. | Page 9-14, Figure 2-5 |
|  | 20d | Present results of all sensitivity analyses conducted to assess the robustness of the synthesized results. | Page 14 |
| Reporting biases | 21 | Present assessments of risk of bias due to missing results (arising from reporting biases) for each synthesis assessed. | Page 14 |
| Certainty of evidence | 22 | Present assessments of certainty (or confidence) in the body of evidence for each outcome assessed. | Not applicable |
| **DISCUSSION** | | |  |
| Discussion | 23a | Provide a general interpretation of the results in the context of other evidence. | Page 14-15 |
|  | 23b | Discuss any limitations of the evidence included in the review. | Page 19-20 |
|  | 23c | Discuss any limitations of the review processes used. | Page 19 |
|  | 23d | Discuss implications of the results for practice, policy, and future research. | Page 17-19 |
| **OTHER INFORMATION** | | |  |
| Registration and protocol | 24a | Provide registration information for the review, including register name and registration number, or state that the review was not registered. | Page 5 |
|  | 24b | Indicate where the review protocol can be accessed, or state that a protocol was not prepared. | Page 6 |
|  | 24c | Describe and explain any amendments to information provided at registration or in the protocol. | No modifications |
| Support | 25 | Describe sources of financial or non-financial support for the review, and the role of the funders or sponsors in the review. | Page 21 |
| Competing interests | 26 | Declare any competing interests of review authors. | Page 21 |
| Availability of data, code and other materials | 27 | Report which of the following are publicly available and where they can be found: template data collection forms; data extracted from included studies; data used for all analyses; analytic code; any other materials used in the review. | Page 22 |

*From:*  Page MJ, McKenzie JE, Bossuyt PM, Boutron I, Hoffmann TC, Mulrow CD, et al. The PRISMA 2020 statement: an updated guideline for reporting systematic reviews. BMJ 2021;372:n71. doi: 10.1136/bmj.n71

For more information, visit: <http://www.prisma-statement.org/>

Table S2. Characteristics of included studies in this systematic review and meta-analysis

| **No.** | **Study ID ^a^** | **Country** | **Study design** | **Vaccination status** | **Vaccine product** | **SARS-CoV-2 Variant** | **Clinical outcomes** | **Study population** | **Sample size** | **NOS score** |
| --- | --- | --- | --- | --- | --- | --- | --- | --- | --- | --- |
| 1 | Peter J. Embi(1) | The USA | Test-negative case-control study | Full | BNT162b2 & mRNA-1273 | Delta | 1. COVID-19 related hospitalization | Immunocompetent and immunocompromised adults aged 18 years and older | Cases: 12498 Controls: 76719 | 9 |
| 2 | Yinong Young-Xu(2) | The USA | Case-control study | Full | BNT162b2 & mRNA-1273 | Delta | 1.Laboratory-confirmed infection | The elder (Veterans aged 65 years or older) | Cases: 14238 Controls: 56952 | 8 |
| 3 | Sara S Kim(3) | The USA | Test-negative case-control study | Full & Partial | BNT162b2 & mRNA-1273 | Alpha | 1. PCR-confirmed infection | General population (Aged 16 years or older) | Cases: 236 Controls: 576 | 7 |
| 4 | Brechje de Gier(4) | Netherlands | Cohort study | Full & Partial | BNT162b2 & mRNA-1273 & ChAdOx1 nCoV-19 & Ad26.COV2.S | Alpha | 1. PCR-confirmed infection | SARS-CoV-2-positive contacts | No vaccination: 243360 Partial vaccination: 4411 Full vaccination: 5397 | 7 |
| 5 | Elina Seppälä(5) | Norway | Cohort study | Full & Partial | BNT162b2 & mRNA-1273 | Alpha & Delta | 1.PCR or whole genome sequencing confirmed infection | General population (Adults aged 18 years or older) | No vaccination: 909175 Partial vaccination: 1360772 Full vaccination: 1934912 | 7 |
| 6 | Aharona Glatman-Freedman(6) | Israel | Cohort study | Full | BNT162b2 | Delta | 1. PCR-confirmed infection | Adolescents (Aged 12-15 years) | No vaccination & Partial vaccination: 413918 Full vaccination: 187707 | 7 |
| 7 | Benjamin Lefèvre(7) | France | Cohort study | Full & Partial | BNT162b2 | Beta | 1.PCR-confirmed infection  2. COVID-19 related severe disease (COVID-19 related hospitalization and death) | Residents of long-term care facilities | No vaccination: 290 Partial vaccination: 17 Full vaccination: 66 | 8 |
| 8 | Karen Lutrick(8) | The USA | Cohort study | Full | BNT162b2 | Delta | 1. PCR-confirmed infection | Adolescents (Aged 12-17 years) | No vaccination: 66 Partial vaccination: 30 Full vaccination: 190 | 8 |
| 9 | Mark A. Katz(9) | Israel | Cohort study | Full | BNT162b2 | Alpha | 1. PCR-confirmed infection | Healthcare Workers | No vaccination: 252  Full vaccination: 998 | 7 |
| 10 | Laith J. Abu-Raddad(10) | Qatar | Test-negative case-control study & Cohort study | Full & Partial | BNT162b2 | Alpha & Beta | 1. PCR-confirmed infection 2. Severe, critical or fatal COVID-19 disease | General population (Residents population in Qatar) | Cases: 78044 Controls: 78044 | 8 |
| 11 | Shepherd R. Singer(11) | Israel | Cohort study | Full & Partial | BNT162b2 | Beta | 1. PCR-confirmed infection 2. COVID-19 related hospitalization 3. COVID-19 related death | SARS-CoV-2-positive contacts | No vaccination: 248 Partial vaccination: 33 Full vaccination: 33 | 8 |
| 12 | Khitam Muhsen(12) | Israel | Cohort study | Full | BNT162b2 | Alpha | 1. PCR-confirmed infection | Healthcare Workers | No vaccination: 2202 Full vaccination: 6960 | 8 |
| 13 | Aziz Sheikh(13) | The UK | Cohort study | Full & Partial | BNT162b2 & ChAdOx1 nCoV-19 | Delta | 1. COVID-19 related death | General population (Adults aged 18 years or older) | No vaccination: 40233 Partial & Full vaccination:62246 | 8 |
| 14 | Walid Q. Alali(14) | Kuwait | Cohort study | Full & Partial | BNT162b2 & ChAdOx1 nCoV-19 | Alpha | 1. PCR-confirmed infection | Healthcare Workers | No vaccination: 2217 Partial vaccination: 108  Full vaccination: 921 | 6 |
| 15 | Hannah Chung(15) | Canada | Test-negative case-control study | Full & Partial | BNT162b2 & mRNA-1273 | Alpha, Beta & Gamma | 1. PCR-confirmed infection 2. COVID-19 related severe disease (COVID-19 related hospitalization and death) | General population (Aged 16 years or older) | Cases: 42567 Controls: 281466 | 9 |
| 16 | Patrick Tang(16) | Qatar | Test-negative case-control study | Full & Partial | BNT162b2 & mRNA-1273 | Delta | 1. PCR-confirmed infection 2. Severe, critical or fatal COVID-19 | General population (Residents population in Qatar) | Cases: 4683 Controls: 720720 | 9 |
| 17 | Shirley Collie(17) | South Africa | Test-negative case-control study | Full | BNT162b2 | Omicron | 1. COVID-19 related hospitalization | General population (Adults aged 18 years or older) | Cases: 429 Controls: 77744 | 7 |
| 18 | E. Sansone(18) | Italy | Cohort study | Full | BNT162b2 | Alpha | 1. PCR-confirmed infection | Healthcare Workers | No vaccination: 52 Full vaccination: 40 | 7 |
| 19 | Ben Y. Reis(19) | Israel | Cohort study | Full & Partial | BNT162b2 | Delta | 1. PCR-confirmed infection | Adolescents (Aged 12-18 years) | No vaccination: 94354 Full vaccination: 94354 | 8 |
| 20 | Emanuele Sansone(20) | Italy | Case-control study | Full | BNT162b2 | Alpha | 1. PCR-confirmed infection | Healthcare Workers | Cases: 92 Controls: Not Mentioned | 6 |
| 21 | Ramachandran Thiruvengadam(21) | India | Test-negative case-control study | Full & Partial | ChAdOx1 nCoV-19 | Delta | 1. PCR-confirmed infection 2. Moderate-to-severe COVID-19 disease | Healthcare Workers | Cases: 2766 Controls: 2377 | 9 |
| 22 | Matt D.T. Hitchings(22) | Brazil | Test-negative case-control study | Full & Partial | ChAdOx1 nCoV-19 | Gamma | 1. PCR-confirmed infection 2. COVID-19 related hospitalization 3. COVID-19 related ICU admission 4. COVID-19-related death | The elder (Adults aged 60 years or older) | Cases: 30680 Controls: 30680 | 9 |
| 23 | Otavio T Ranzani(23) | Brazil | Test-negative case-control study | Full & Partial | CoronaVac | Gamma | 1. PCR-confirmed infection 2. COVID-19 related hospitalization 3. COVID-19 related death | The elder (Adults aged 70 years or older) | Cases: 13283 Controls: 42236 | 9 |
| 24 | Kristina L. Bajema(24) | The USA | Test-negative case-control study | Full | BNT162b2 & mRNA-1273 | Delta | 1. COVID-19 related hospitalization | General population (Adults aged 18 years or older) | Cases: 388 Controls: 787 | 7 |
| 25 | Ashley Fowlkes(25) | The USA | Cohort study | Full | BNT162b2 & mRNA-1273 | Delta | 1. PCR-confirmed infection | Healthcare Workers | No vaccination: 488  Full vaccination: 2352 | 8 |
| 26 | Mark G. Thompson(26) | The USA | Test-negative case-control study | Full & Partial | BNT162b2 & mRNA-1273 & Ad26.COV2.S | Alpha | 1. COVID-19 related hospitalization 2. COVID-19 related ICU admission 3.COVID-19 related Emergency Department or Urgent Care Clinic Visit | Hospitalizations of patients with Covid-19–like illness (Adults aged 50 years or older) | Cases: 7572 Controls: 55502 | 9 |
| 27 | Iván Martínez-Baz(27) | Spain | Cohort study | Full & Partial | BNT162b2 & mRNA-1273 & ChAdOx1 nCoV-19 | Alpha | 1. PCR-confirmed infection 2. COVID-19 related hospitalization | SARS-CoV-2-positive contacts | No vaccination: 19580 Partial vaccination: 869 Full vaccination: 512 | 7 |
| 28 | Jamie Lopez Bernal(28) | The UK | Test-negative case-control study | Full & Partial | BNT162b2 & ChAdOx1 nCoV-19 | Alpha, Delta | 1. PCR-confirmed infection | General population (Aged 16 years or older) | Cases: 15558 Controls: 171834 | 9 |
| 29 | Nick Andrews(29) | The UK | Test-negative case-control study | Full & Partial | BNT162b2 & mRNA-1273 &ChAdOx1 nCoV-19 | Alpha, Delta | 1. PCR-confirmed infection 2. COVID-19 related hospitalization 3. COVID-19-related death | General population (Aged 16 years or older) | Cases: 544468 Controls: 4349930 | 9 |
| 30 | Oon Tek Ng(30) | Singapore | Cohort study | Full | BNT162b2 & mRNA-1273 | Delta | 1. PCR-confirmed infection | SARS-CoV-2-positive contacts | No vaccination: 27135 Partial & Full vaccination: 716391 | 8 |
| 31 | Koen B. Pouwels(31) | The UK | Test-negative case-control study | Full & Partial | BNT162b2 & ChAdOx1 nCoV-19 | Alpha, Delta | 1. PCR-confirmed infection | General population (Adults aged 18 years or older) | No vaccination: 27135 Partial & Full vaccination: 716391 | 9 |
| 32 | Sara Y Tartof(32) | The USA | Cohort study | Full | BNT162b2 | Delta | 1. PCR-confirmed infection 2. COVID-19 related hospitalization | General population (Aged 12 years or older) | No vaccination: 2290189 Partial & Full vaccination:1146768 | 9 |
| 33 | Baltazar Nunes(33) | Portugal | Cohort study | Full & Partial | BNT162b2 & mRNA-1273 | Alpha | 1. COVID-19 related hospitalization 2. COVID-19 related death | The elder (Adults aged 65 years or older) | No vaccination: 125338 Partial & Full vaccination: 753151 | 7 |
| 34 | Mark W. Tenforde(34) | The USA | Case-control study | Full | BNT162b2 & mRNA-1273 | Alpha, Delta | 1. COVID-19 related hospitalization 2. COVID-19-related death | General population (Adults aged 18 years or older) | Cases: 1983 Controls: 2530 | 9 |
| 35 | Hiam Chemaitelly(35) | Qatar | Test-negative case-control study | Full & Partial | mRNA-1273 | Alpha, Beta | 1. PCR-confirmed infection 2. Severe, critical or fatal COVID-19 disease | General population (Resident population of Qatar) | Cases: 4497 Controls: 4497 | 9 |
| 36 | Katia J Bruxvoort(36) | The USA | Test-negative case-control study | Full & Partial | mRNA-1273 | Alpha, Gamma and Delta | 1. PCR-confirmed infection | General population (Adults aged 18 years or older) | Cases: 10135 Controls: 2027 | 9 |
| 37 | Christophe Paris(37) | France | Cohort study | Partial | BNT162b2 & mRNA-1273 & ChAdOx1 nCoV-19 | Alpha | 1. PCR-confirmed infection | Healthcare Workers | No vaccination: 3573 Partial & Full vaccination: 4593 | 8 |
| 38 | Laith J. Abu-Raddad^#(38)^ | Qatar | Test-negative case-control study | Partial | BNT162b2 | Alpha, Beta | 1. PCR-confirmed infection 2. Severe, critical or fatal COVID-19 disease | General population (Resident population in Qatar) | No vaccination: 486 Partial vaccination: 36780 | 7 |
| 39 | Samantha M. Olson(39) | The USA | Test-negative case-control study | Full | BNT162b2 | Delta | 1. COVID-19 related hospitalization | Adolescents (Aged 12-18 years) | Cases: 179 Controls: 285 | 8 |
| 40 | Jamie Lopez Bernal^#(40)^ | The UK | Test-negative case-control study | Full & Partial | BNT162b2 & ChAdOx1 nCoV-19 | Alpha | 1. PCR-confirmed infection 2. COVID-19 related hospitalization 3. COVID-19-related death | The elder (Aged 70 years or older) | Cases: 44590 Controls: 108851 | 9 |
| 41 | Shaun J. Grannis(41) | The USA | Test-negative case-control study | Full | BNT162b2 & mRNA-1273 & Ad26.COV2.S | Delta | 1. COVID-19 related hospitalization 2. COVID-19 associated emergency department and urgent care clinic encounters | General population (Adults aged 18 years or older) | Cases: 1551 Controls: 13085 | 7 |
| 42 | Rebecca Grant(42) | France | Case-control study | Full & Partial | BNT162b2 & ChAdOx1 nCoV-19 | Delta | 1. PCR-confirmed infection | General population (Adults aged 18 years or older) | Cases: 12634 Controls: 5560 | 8 |
| 43 | Francesca Rovida(43) | Italy | Cohort study | Full | BNT162b2 | Alpha | 1. PCR-confirmed infection | Healthcare Workers | No vaccination: 3720 Full vaccination: 346 | 9 |
| 44 | Iván Martínez-Baz^#(44)^ | Spain | Cohort study | Full & Partial | BNT162b2 & mRNA-1273 & ChAdOx1 nCoV-19 | Alpha, Delta | 1. PCR-confirmed infection | SARS-CoV-2-positive contacts | No vaccination: 14348 Partial vaccination: 4138 Full vaccination: 11754 | 7 |
| 45 | Eric J Haas(45) | Israel | Cohort study | Full | BNT162b2 | Alpha | 1. PCR-confirmed infection 2. COVID-19 related hospitalizations 3. COVID-19 related Severe or critical hospitalizations 4. COVID-19 related deaths | General population (Aged 16 years or older) | No vaccination: 1823979 Full vaccination: 4714932 | 9 |
| 46 | Noa Dagan(46) | Israel | Cohort study | Full & Partial | Pfizer–BioNTech | Alpha | 1. PCR-confirmed infection 2. COVID-19 related hospitalizations 3. COVID-19 related Severe or critical hospitalizations 4. COVID-19 related death | General population (Aged 16 years or older) | No vaccination: 596618 Full vaccination: 596618 | 9 |
| 47 | Lindsay T. Keegan(47) | The USA | Cohort study | Full | BNT162b2 & mRNA-1273 & Ad26.COV2.S | Delta | 1. PCR-confirmed infection | General population (Aged 12 years or older) | No vaccination: 1479012 Full vaccination: 1726946 | 8 |
| 48 | Esther Kissling(48) | Eight European countries | Test-negative case-control study | Full & Partial | BNT162b2 &ChAdOx1 nCoV-19 | Alpha | 1. PCR-confirmed infection | The elder (Adults aged 65 years or older) | Cases: 592 Controls: 4372 | 8 |
| 49 | Zoltán Vokó(49) | Hungary | Cohort study | Full | BNT162b2 & mRNA-1273 & Ad26.COV2.S & Gam-COVID-Vac & BBIBP-CorV | Alpha | 1. PCR or viral genome sequencing confirmed infection 2. COVID-19 related Deaths | General population (Aged 16 years or older) | No vaccination:  Full vaccination: 3740066 | 8 |
| 50 | Sara Carazo(50) | Canada | Test-negative case-control study | Full & Partial | BNT162b2 & mRNA-1273 | Alpha | 1. PCR-confirmed infection | Healthcare Workers | Cases: 5316 Controls: 53160 | 9 |
| 51 | Danuta M Skowronski(51) | Canada | Test-negative case-control study | Partial | BNT162b2 & mRNA-1273 | Alpha, Gamma | 1. PCR-confirmed infection | The elder (Adults aged 70 years or older) | Cases: 1226 Controls: 15767 | 9 |
| 52 | Hiam Chemaitelly^#(52)^ | Qatar | Test-negative case-control study | Full & Partial | BNT162b2 | Alpha, Beta and Delta | 1. PCR-confirmed infection 2. COVID-19 related hospitalizations 3. COVID-19 related severe or critical hospitalizations 4. COVID-19 related death | General population (Aged 12 years or older) | Cases: 142300 Controls: 848240 | 9 |
| 53 | Devashish Desai(53) | India | Test-negative case-control study | Full & Partial | BBV152 | Delta | 1. PCR-confirmed infection | Healthcare Workers | Cases: 1068 Controls: 1068 | 9 |
| 54 | Tiffany Charmet(54) | France | Case-control study | Full | BNT162b2 & mRNA-1273 | Alpha, Beta and Gamma | 1. PCR-confirmed infection | General population (Adults aged 18 years or older) | Cases: 33863 Controls: 3644 | 9 |
| 55 | Ruma Satwik(55) | India | Cohort study | Full & Partial | ChAdOx1 nCoV-19 | Delta | 1. PCR-confirmed infection 2. COVID-19 related Moderate to Severe Disease  3. Need for oxygen therapy  4. COVID-19 related deaths | Healthcare Workers | No vaccination: 927 Partial vaccination: 623 Full vaccination: 2176 | 7 |
| 56 | Maria Elena Flacco(56) | Italy | Cohort study | Full & Partial | BNT162b2 & mRNA-1273 & ChAdOx1 nCoV-19 | Alpha | 1. PCR-confirmed infection 2. COVID-19 related hospitalization 3. COVID-19-related death | General population (Adults aged 18 years or older) | No vaccination: 175687 Partial & Full vaccination: 69539 | 8 |
| 57 | Thiago Cerqueira-Silva(57) | Brazil | Cohort study | Full & Partial | CoronaVac & ChAdOx1 nCoV-19 | Gamma | 1. PCR-confirmed infection 2. COVID-19 related hospitalization 3. COVID-19 related ICU admission 4. COVID-19-related death | General population (Adults aged 18 years or older) | No vaccination: Not Mentioned Partial & Full vaccination: 75919840 | 8 |
| 58 | Annalee Yassi(58) | Canada | Cohort study | Full & Partial | BNT162b2 & mRNA-1273 | Gamma | 1. PCR-confirmed infection | Healthcare Workers | No vaccination: 3440 Partial & Full vaccination: 22118 | 7 |
| 59 | Paul Elliott(59) | The UK | Cohort study | Full | BNT162b2 & mRNA-1273 | Delta | 1. PCR-confirmed infection | General population (Adults aged 18-64 years) | No vaccination: 1553 Partial vaccination: 13170 Full vaccination: 34930 | 7 |
| 60 | Lamprini Veneti(60) | Norway | Cohort study | Full & Partial | BNT162b2 & mRNA-1273 | Alpha, Delta | 1. COVID-19 related hospitalization | General population (All residents in Norway) | No vaccination: 15140 Partial vaccination: 2386  Full vaccination: 807 | 8 |
| 61 | Marta Grgič Vitek(61) | Slovenia | Cohort study | Full | BNT162b2 & mRNA-1273 & ChAdOx1 nCoV-19 | Delta | 1. COVID-19 related hospitalization | General population (Adults aged 18 years or older) | No vaccination: 371 Full vaccination: Not Mentioned | 7 |
| 62 | Xiao-Ning Li(62) | China | Test-negative case-control study | Full & Partial | CoronaVac | Delta | 1. Different clinical severities(1. mild 2. moderate 3. severe 4. critical illness) | SARS-CoV-2-positive close contacts | Cases: 74 Controls: 292 | 7 |
| 63 | Elizabeth T. Chin(63) | The USA | Cohort study | Full | mRNA-1273 | Delta | 1. PCR-confirmed infection | Residents in prison | No vaccination: 359 Full vaccination: 468 | 8 |
| 64 | Eli S. Rosenberg(64) | The USA | Cohort study | Full | BNT162b2 & mRNA-1273 & Ad26.COV2.S | Delta | 1. Laboratory-Confirmed infections | General population (Adults aged 18 years or older) | No vaccination: 3052683 Full vaccination: 5638142 | 9 |
| 65 | Mark W. Tenforde^#(65)^ | The USA | Test-negative case-control study | Full | BNT162b2 & mRNA-1273 | Alpha | 1. COVID-19 related hospitalization | General population (Adults aged 18 years or older) | Cases: 593 Controls: 619 | 8 |
| 66 | Ulrike Baum(66) | Finnish | Cohort study | Full & Partial | BNT162b2 & mRNA-1273 & ChAdOx1 nCoV-19 | Alpha | 1. PCR-confirmed infection 2. COVID-19 related hospitalization | The elder (Adults aged 70 years or older) | No vaccination: 339224 Partial & Full vaccination: 1336394 | 7 |
| 67 | Aziz Sheikh^#(67)^ | The UK | Test-negative case-control study | Full & Partial | BNT162b2 & ChAdOx1 nCoV-19 | Alpha, Delta | 1. PCR-confirmed infection | General population (Aged 15 years or older) | Not Mentioned | 8 |
| 68 | Victoria Jane Hall(68) | The UK | Cohort study | Full & Partial | BNT162b2 & ChAdOx1 nCoV-19 | Alpha | 1. PCR-confirmed infection | Healthcare Workers | No vaccination: 2683 Partial & Full vaccination: 20641 | 9 |
| 69 | Brechje de Gier^#(69)^ | Netherlands | Cohort study | Full & Partial | BNT162b2 & mRNA-1273 & ChAdOx1 nCoV-19 & Ad26.COV2.S | Delta | 1. PCR, loop mediated isothermal amplification (LAMP) or antigen test-confirmed infection | SARS-CoV-2-positive contacts | No vaccination: 2941 Partial vaccination: 641 Full vaccination: 4189 | 8 |
| 70 | Aakashneel Bhattacharya(70) | India | Case-control study | Full | BBV152 | Delta | 1. PCR-confirmed infection  2. COVID-19 related hospitalization 3. COVID-19 related ICU admission 4. COVID-19-related death | General population (Adults aged 18 years or older) | Cases: 292 Controls: 346 | 8 |
| 71 | Srinivas Nanduri(71) | The USA | Cohort study | Full | BNT162b2 & mRNA-1273 | Delta | 1. PCR-confirmed infection | Nursing home and long-term care facility residents | No vaccination: 953861 Full vaccination: 5011746 | 8 |
| 72 | Mark W. Tenforde*(72) | The USA | Case-control study | Full | BNT162b2 & mRNA-1273 | Delta | 1. COVID-19 related hospitalization | General population (Adults aged 18 years or older) | Cases: 1194 Controls: 1895 | 8 |
| 73 | Srinivasa Vittal Katikireddi(73) | The UK, Brazil | Cohort study | Full & Partial | ChAdOx1 nCoV-19 | Gamma, Delta | 1. PCR-confirmed infection  2. COVID-19 related hospitalization 3. COVID-19-related death | General population (Adults aged 18 years or older) | No vaccination: 9878183 Partial vaccination: 1159137 Full vaccination: 495665 | 9 |
| 74 | Madhumita Shrotri(74) | The UK | Cohort study | Partial | BNT162b2 & ChAdOx1 nCoV-19 | Alpha | 1. PCR-confirmed infection | Long-term care facilities (Residents aged 65 years and older ) | No vaccination: 1252 Partial vaccination: 9160 | 9 |
| 75 | Catherine Hyams(75) | The UK | Test-negative case-control study | Partial | BNT162b2 & ChAdOx1 nCoV-19 | Alpha | 1. PCR-confirmed infection | The elder (Adults aged 80 years or older) | Cases: 144 Controls: 322 | 9 |
| 76 | Matt D.T. Hitchings^#(76)^ | Brazil | Test-negative case-control study | Full & Partial | CoronaVac | Gamma | 1. PCR-confirmed infection | Healthcare Workers | Cases: 811 Controls: 811 | 9 |
| 77 | Emma K. Accorsi(77) | The USA | Test-negative case-control study | Booster | BNT162b2 & mRNA-1273 | Delta, Omicron | 1. PCR-confirmed infection | General population (Adults aged 18 years or older) | Cases: 23391  Controls: 46764 | 8 |
| 78 | Massimo Fabiani(78) | Italy | Cohort study | Booster | BNT162b2 & mRNA-1273 | Delta | 1. PCR-confirmed infection  2. COVID-19 related severe illness (COVID-19 related hospitalization or death) | 1. The elder (Adults aged 70 years or older) 2. Healthcare Workers | No vaccination: Not Mentioned Partial & Full vaccination: 18524568 | 8 |
| 79 | Young June Choe(79) | South Korea | Cohort study | Full & Partial | BNT162b2 | Delta | 1. PCR-confirmed infection | Adolescents (Aged 16-18 years) | No vaccination: Not Mentioned Partial & Full vaccination: 883401 | 6 |
| 80 | Amadea Britton(80) | The USA | Test-negative case-control study | Full | BNT162b2 & mRNA-1273 & ChAdOx1 nCoV-19 | Delta | 1. PCR-confirmed infection | 1. General population (Adults aged 20 years or older) 2. Adolescents (Aged 12-19 years) | Cases: 390762 Controls: 1423621 | 9 |
| 81 | Massimo Fabiani^#(81)^ | Italy | Cohort study | Full & Partial | BNT162b2 & mRNA-1273 | Alpha, Delta | 1. PCR-confirmed infection  2. COVID-19 related severe illness | 1. General population (Aged 16 years or older) 2. Healthcare Workers | No vaccination: Not Mentioned Partial & Full vaccination: 33250344 | 9 |
| 82 | Mingshuang Li(82) | China | Case control study | Full & Partial & Booster | Adenovirus-vectored vaccine (CoronaVac & BBIBP-CorV) | Delta, Omicron | 1. Different clinical symptoms (Asymptomatic, mild, moderate, serious, or severe) | 1. General population (Aged 3 years or older) | Cases: 10829  Controls: Not Mentioned | 6 |
| 83 | Jill M. Ferdinands(84) | The USA | Test-negative case-control study | Full & Booster | BNT162b2 & mRNA-1273 | Delta, Omicron | 1. COVID-19 emergency department/urgent care (ED/UC) visits 2. COVID-19 related hospitalization | General population (Adults aged 18 years or older) | Cases: 61826 Controls: 179378 | 9 |
| 84 | Tommy Nyberg(85) | The UK | Cohort study | Full & Partial & Booster | BNT162b2 & mRNA-1273 & ChAdOx1 nCoV-19 | Delta, Omicron | 1. COVID-19 related hospitalization 2. COVID-19-related death | General population (Residents population in The UK) | Cases: 1516702 Controls: Not Mentioned | 9 |
| 85 | Huong Q McLean(86) | The USA | Cohort study | Full | BNT162b2 & mRNA-1273 | Delta | 1. PCR-confirmed infection | General population (Aged 12 years or older) | No vaccination: 329 Full vaccination: 937 | 6 |
| 86 | Jessie R Chung(87) | The USA | Test-negative case-control study | Full | BNT162b2 & mRNA-1273 | Delta | 1. PCR-confirmed infection | General population (Aged 12 years or older) | Cases: 614 Controls: 1615 | 7 |
| 87 | Stuti Pramod(88) | India | Test-negative case-control study | Full & Partial | Covishield | Delta | 1. PCR-confirmed infection 2. COVID-19 related ICU admission | Healthcare Workers | Cases: 360 Controls: 360 | 7 |
| 88 | Hung Fu Tseng(89) | The USA | Test-negative case-control study | Booster & Full & Partial | mRNA-1273 | Delta, Omicron | 1. PCR-confirmed infection 2. COVID-19 related hospitalization | General population (Adults aged 18 years or older) | Cases: 26683 Controls: 109662 | 9 |
| 89 | Paul M McKeigue(90) | The UK | Case-control study | Full | BNT162b2 & mRNA-1273 &ChAdOx1 nCoV-19 | Delta | 1. COVID-19 related Severe illness | General population | Cases: 5644 Controls: 21671 | 9 |
| 90 | Zhiliang Hu(91) | China | Case-control study | Full & Partial | CoronaVac | Delta | 1. COVID-19 related Severe illness | General population (Adults aged 18 years or older) | Cases: 476 Controls: Not Mentioned | 7 |
| 91 | Jennifer M. Polinski(92) | The USA | Cohort study | Full | Ad26.COV2.S | Delta | 1. PCR-confirmed infection 2. COVID-19 related hospitalization | General population (Adults aged 18 years or older) | No vaccination: 422034 Full vaccination: 1645397 | 9 |
| 92 | Mie Agermose Gram(93) | Danmark | Cohort study | Full & Partial | BNT162b2 & mRNA-1273 & ChAdOx1 nCoV-19 | Alpha | 1. PCR-confirmed infection 2. COVID-19 related hospitalization | General population (Resident population of Danmark) | No vaccination: 273298 Partial & Full vaccination: 280911 | 7 |
| 93 | Monica M. Robles Fontán(94) | The USA | Cohort study | Full | BNT162b2 & mRNA-1273 & Ad26.COV2.S | Delta | 1. PCR-confirmed infection | General population (Aged 12 years or older) | Not Mentioned | 9 |
| 94 | Sara Y Tartof^#(95)^ | The USA | Test-negative case-control study | Booster & Full | BNT162b2 | Delta, Omicron | 1. COVID-19 Emergency department 2. COVID-19 related hospitalization | General population (Adults aged 18 years or older) | Delta Cases: 2838 Omicron Cases: 4523 Controls: 3762 | 9 |
| 95 | Nicola P. Klein(96) | The USA | Test-negative case-control study | Booster & Full | BNT162b2 | Delta, Omicron | 1. COVID-19 Emergency department 2. COVID-19 related hospitalization | Adolescents (Aged 5-17 years) | Cases: 29965 Controls: 9252 | 8 |
| 96 | Ashley M. Price(97) | The USA | Test-negative case-control study | Full | BNT162b2 | Delta, Omicron | 1. COVID-19 related hospitalization 2. COVID-19 related severe illness(COVID-19 related hospitalization or death) | Adolescents (Aged 12-18 years) | Cases: 29965 Controls: 1627 | 9 |
| 97 | Alejandro Jara(98) | Chile | Cohort study | Booster | CoronaVac & BNT162b2 & Ad26.COV2.S | Delta | 1. PCR-confirmed infection  2. COVID-19 related hospitalization 3. COVID-19 related ICU admission 4. COVID-19-related death | General population (Aged 16 years or older) | No vaccination: 2889319 Booster vaccination: 4127546 | 9 |
| 98 | Mark W. Tenforde^(99) | The USA | Case-control study | Booster & Full | BNT162b2 & mRNA-1273 & ChAdOx1 nCoV-19 | Delta, Omicron | 1.COVID-19 related severe illness(Invasive Mechanical Ventilation and Death) | General population (Adults aged 18 years or older) | Cases: 1440 Controls: 6104 | 8 |
| 99 | Karthik Natarajan(100) | The USA | Test-negative case-control study | Booster & Full | BNT162b2 & mRNA-1273 & Ad26.COV2.S | Omicron | 1. COVID-19 Emergency department 2. COVID-19 related hospitalization | General population (Adults aged 18 years or older) | Cases: 28127 Controls: 52160 | 8 |
| 100 | Milena Suarez Castillo(101) | France | Test-negative case-control study | Booster & Full | BNT162b2 & mRNA-1273 & ChAdOx1 nCoV-19 | Delta, Omicron | 1. PCR-confirmed infection | General population (Adults aged 18 years or older) | Cases: 926376 Controls: 1852752 | 8 |
| 101 | Ashley L. Fowlkes(102) | The USA | Cohort study | Booster & Full | BNT162b2 | Delta, Omicron | 1. PCR-confirmed infection | Adolescents (Aged 5-15 years) | No vaccination: 386 Full vaccination & Booster: 978 | 8 |
| 102 | Aditya Sharma(103) | Malaysia | Cohort study | Booster & Full | BNT162b2 & mRNA-1273 | Omicron | 1. PCR-confirmed infection  2. COVID-19 related hospitalization 3. COVID-19-related death | General population (Adults aged 18 years or older) | No vaccination: 408774 Full vaccination: 408774 Booster vaccination: 408774 | 8 |
| 103 | Nick Andrews#(104) | The UK | Test-negative case-control study | Booster & Full & Partial | BNT162b2 & ChAdOx1 nCoV-19 | Delta, Omicron | 1. PCR-confirmed infection | General population (Aged 16 years or older) | Delta Cases: 204154 Omicron Cases: 886774 Controls: 1572621 | 9 |
| 104 | Adam S Lauring(105) | The USA | Test-negative case-control study | Booster & Full & Partial | BNT162b2 & mRNA-1273 | Alpha, Delta and Omicron | 1. COVID-19 related hospitalization | General population (Adults aged 18 years or older) | Cases: 5728 Controls: 5962 | 9 |
| 105 | Mark G. Thompson^#^(106) | The USA | Test-negative case-control study | Booster & Full & Partial | BNT162b2 & mRNA-1273 & Ad26.COV2.S | Delta, Omicron | 1. COVID-19 related hospitalization 2. COVID-19 related Emergency Department or Urgent Care Clinic Visit | General population (Adults aged 18 years or older) | Cases: 36542 Controls: 61545 | 8 |
| 106 | Paskorn Sritipsukho(107) | Thailand | Test-negative case-control study | Booster & Full & Partial | CoronaVac & BNT162b2 & ChAdOx1 nCoV-19 | Delta | 1. Different clinical severities (1. mild 2. moderate 3. severe 4. critical illness) | General population (Adults aged 18 years or older) | Cases: 12 Controls: 478 | 8 |
| 107 | Emma K. Accorsi^#^(108) | The USA | Test-negative case-control study | Booster | mRNA-1273 & BNT162b2 & Ad26.COV2.S | Omicron | 1. PCR-confirmed infection | General population (Adults aged 18 years or older) | Cases: 187804 Controls: 325124 | 9 |
| 108 | Thiago Cerqueira-Silva^#^(109) | Brazil | Test-negative case-control study | Booster & Full & Partial | CoronaVac & BNT162b2 | Omicron | 1. PCR-confirmed infection  2. COVID-19 related severe illness(COVID-19 related hospitalization or death) | General population (Adults aged 18 years or older) | Cases: 210856 Controls: 130560 | 9 |
| 109 | Aaron Richterman(110) | The USA | Test-negative case-control study | Booster & Full | mRNA-1273 & BNT162b2 | Omicron | 1. PCR-confirmed infection | General population (Adults aged 18 years or older) | Cases: 2776 Controls: 11744 | 9 |
| 110 | Hiam Chemaitelly*(111) | Qutar | Test-negative case-control study | Booster & Full & Partial | BNT162b2 | Omicron | 1. PCR-confirmed infection  2. COVID-19 related severe illness(COVID-19 related hospitalization or death) | General population (Adults aged 18 years or older) | Cases: 39855 Controls: 23814 | 9 |
| 111 | Heba N. Altarawneh(112) | Qutar | Test-negative case-control study | Booster | BNT162b2 | Omicron | 1. PCR-confirmed infection  2. COVID-19 related severe illness(COVID-19 related hospitalization or death) | General population (Adults aged 18 years or older) | Cases: 15267 Controls: 14186 | 9 |
| 112 | Freja CM Kirsebom(113) | The UK | Test-negative case-control study | Booster | mRNA-1273 & BNT162b2 &ChAdOx1 nCoV-19 | Omicron | 1. PCR-confirmed infection  2. COVID-19 related hospitalization | General population (Adults aged 18 years or older) | Cases: 511889 Controls: 615628 | 9 |
| 113 | Martina E McMenamin(114) | China | Cohort study | Booster & Full & Partial | CoronaVac & BNT162b2 | Omicron | 1. Different clinical symptoms (Mild, moderate, severe or fatal) | General population (Adults aged 20 years or older) | Unvaccinated: 13019 Vaccinated: 8288 | 9 |

^a^ We used the first author of the paper as the ID of the study. If the author was found to have published more than one study in the study we included, we used the “#” in the upper right corner to represent the second study published by the author, “*” for the third time, and “^” for the fourth time.

**Reference:**

1. Embi PJ, Levy ME, Naleway AL, Patel P, Gaglani M, Natarajan K, et al. Effectiveness of 2-Dose Vaccination with mRNA COVID-19 Vaccines Against COVID-19-Associated Hospitalizations Among Immunocompromised Adults - Nine States, January-September 2021. MMWR Morb Mortal Wkly Rep. 2021 Nov 5;70(44):1553-9.

2. Young-Xu Y, Zwain GM, Powell EI, Smith J. Estimated Effectiveness of COVID-19 Messenger RNA Vaccination Against SARS-CoV-2 Infection Among Older Male Veterans Health Administration Enrollees, January to September 2021. JAMA Netw Open. 2021 Dec 1;4(12):e2138975.

3. Kim SS, Chung JR, Belongia EA, McLean HQ, King JP, Nowalk MP, et al. mRNA Vaccine Effectiveness against COVID-19 among Symptomatic Outpatients Aged >/=16 Years in the United States, February - May 2021. J Infect Dis. 2021 Sep 8.

4. de Gier B, Andeweg S, Joosten R, Ter Schegget R, Smorenburg N, van de Kassteele J, et al. Vaccine effectiveness against SARS-CoV-2 transmission and infections among household and other close contacts of confirmed cases, the Netherlands, February to May 2021. Euro Surveill. 2021 Aug;26(31).

5. Seppala E, Veneti L, Starrfelt J, Danielsen AS, Bragstad K, Hungnes O, et al. Vaccine effectiveness against infection with the Delta (B.1.617.2) variant, Norway, April to August 2021. Euro Surveill. 2021 Sep;26(35).

6. Glatman-Freedman A, Hershkovitz Y, Kaufman Z, Dichtiar R, Keinan-Boker L, Bromberg M. Effectiveness of BNT162b2 Vaccine in Adolescents during Outbreak of SARS-CoV-2 Delta Variant Infection, Israel, 2021. Emerg Infect Dis. 2021 Nov;27(11):2919-22.

7. Lefèvre B, Tondeur L, Madec Y, Grant R, Lina B, van der Werf S, et al. Beta SARS-CoV-2 variant and BNT162b2 vaccine effectiveness in long-term care facilities in France. The Lancet Healthy Longevity. 2021;2(11):e685-e7.

8. Lutrick K, Rivers P, Yoo YM, Grant L, Hollister J, Jovel K, et al. Interim Estimate of Vaccine Effectiveness of BNT162b2 (Pfizer-BioNTech) Vaccine in Preventing SARS-CoV-2 Infection Among Adolescents Aged 12-17 Years - Arizona, July-December 2021. MMWR Morb Mortal Wkly Rep. 2021 Dec 31;70(5152):1761-5.

9. Katz MA, Harlev EB, Chazan B, Chowers M, Greenberg D, Peretz A, et al. Early effectiveness of BNT162b2 Covid-19 vaccine in preventing SARS-CoV-2 infection in healthcare personnel in six Israeli hospitals (CoVEHPI). Vaccine. 2022 Jan 24;40(3):512-20.

10. Abu-Raddad LJ, Chemaitelly H, Butt AA. Effectiveness of the BNT162b2 Covid-19 Vaccine against the B.1.1.7 and B.1.351 Variants. N Engl J Med. 2021 Jul 8;385(2):187-9.

11. Singer SR, Angulo FJ, Swerdlow DL, McLaughlin JM, Hazan I, Ginish N, et al. Effectiveness of BNT162b2 mRNA COVID-19 vaccine against SARS-CoV-2 variant Beta (B.1.351) among persons identified through contact tracing in Israel: A prospective cohort study. EClinicalMedicine. 2021 Dec;42:101190.

12. Muhsen K, Maimon N, Mizrahi A, Bodenneimer O, Cohen D, Maimon M, et al. Effectiveness of BNT162b2 mRNA COVID-19 vaccine against acquisitions of SARS-CoV-2 among health care workers in long-term care facilities: a prospective cohort study. Clin Infect Dis. 2021 Oct 26.

13. Sheikh A, Robertson C, Taylor B. BNT162b2 and ChAdOx1 nCoV-19 Vaccine Effectiveness against Death from the Delta Variant. N Engl J Med. 2021 Dec 2;385(23):2195-7.

14. Alali WQ, Ali LA, AlSeaidan M, Al-Rashidi M. Effectiveness of BNT162b2 and ChAdOx1 Vaccines against Symptomatic COVID-19 among Healthcare Workers in Kuwait: A Retrospective Cohort Study. Healthcare. 2021;9(12).

15. Chung H, He S, Nasreen S, Sundaram ME, Buchan SA, Wilson SE, et al. Effectiveness of BNT162b2 and mRNA-1273 covid-19 vaccines against symptomatic SARS-CoV-2 infection and severe covid-19 outcomes in Ontario, Canada: test negative design study. BMJ. 2021 Aug 20;374:n1943.

16. Tang P, Hasan MR, Chemaitelly H, Yassine HM, Benslimane FM, Al Khatib HA, et al. BNT162b2 and mRNA-1273 COVID-19 vaccine effectiveness against the SARS-CoV-2 Delta variant in Qatar. Nat Med. 2021 Dec;27(12):2136-43.

17. Collie S, Champion J, Moultrie H, Bekker LG, Gray G. Effectiveness of BNT162b2 Vaccine against Omicron Variant in South Africa. N Engl J Med. 2022 Feb 3;386(5):494-6.

18. Sansone E, Tiraboschi M, Sala E, Albini E, Lombardo M, Castelli F, et al. Effectiveness of BNT162b2 vaccine against the B.1.1.7 variant of SARS-CoV-2 among healthcare workers in Brescia, Italy. J Infect. 2021 Jul;83(1):e17-e8.

19. Reis BY, Barda N, Leshchinsky M, Kepten E, Hernán MA, Lipsitch M, et al. Effectiveness of BNT162b2 Vaccine against Delta Variant in Adolescents. N Engl J Med. 2021 Nov 25;385(22):2101-3.

20. Sansone E, Sala E, Tiraboschi M, Albini E, Lombardo M, Indelicato A, et al. Effectiveness of BNT162b2 vaccine against SARS-CoV-2 among healthcare workers. Med Lav. 2021 Jun 15;112(3):250-5.

21. Thiruvengadam R, Awasthi A, Medigeshi G, Bhattacharya S, Mani S, Sivasubbu S, et al. Effectiveness of ChAdOx1 nCoV-19 vaccine against SARS-CoV-2 infection during the delta (B.1.617.2) variant surge in India: a test-negative, case-control study and a mechanistic study of post-vaccination immune responses. The Lancet Infectious Diseases. 2022;22(4):473-82.

22. Hitchings MDT, Ranzani OT, Dorion M, D'Agostini TL, de Paula RC, de Paula OFP, et al. Effectiveness of ChAdOx1 vaccine in older adults during SARS-CoV-2 Gamma variant circulation in Sao Paulo. Nat Commun. 2021 Oct 28;12(1):6220.

23. Ranzani OT, Hitchings MDT, Dorion M, D'Agostini TL, de Paula RC, de Paula OFP, et al. Effectiveness of the CoronaVac vaccine in older adults during a gamma variant associated epidemic of covid-19 in Brazil: test negative case-control study. BMJ. 2021 Aug 20;374:n2015.

24. Bajema KL, Dahl RM, Prill MM, Meites E, Rodriguez-Barradas MC, Marconi VC, et al. Effectiveness of COVID-19 mRNA Vaccines Against COVID-19-Associated Hospitalization - Five Veterans Affairs Medical Centers, United States, February 1-August 6, 2021. MMWR Morb Mortal Wkly Rep. 2021 Sep 17;70(37):1294-9.

25. Fowlkes A, Gaglani M, Groover K, Thiese MS, Tyner H, Ellingson K. Effectiveness of COVID-19 Vaccines in Preventing SARS-CoV-2 Infection Among Frontline Workers Before and During B.1.617.2 (Delta) Variant Predominance - Eight U.S. Locations, December 2020-August 2021. MMWR Morb Mortal Wkly Rep. 2021 Aug 27;70(34):1167-9.

26. Thompson MG, Stenehjem E, Grannis S, Ball SW, Naleway AL, Ong TC, et al. Effectiveness of Covid-19 Vaccines in Ambulatory and Inpatient Care Settings. N Engl J Med. 2021 Oct 7;385(15):1355-71.

27. Martinez-Baz I, Miqueleiz A, Casado I, Navascues A, Trobajo-Sanmartin C, Burgui C, et al. Effectiveness of COVID-19 vaccines in preventing SARS-CoV-2 infection and hospitalisation, Navarre, Spain, January to April 2021. Euro Surveill. 2021 May;26(21).

28. Lopez Bernal J, Andrews N, Gower C, Gallagher E, Simmons R, Thelwall S, et al. Effectiveness of Covid-19 Vaccines against the B.1.617.2 (Delta) Variant. N Engl J Med. 2021 Aug 12;385(7):585-94.

29. Andrews N, Tessier E, Stowe J, Gower C, Kirsebom F, Simmons R, et al. Duration of Protection against Mild and Severe Disease by Covid-19 Vaccines. N Engl J Med. 2022 Jan 27;386(4):340-50.

30. Ng OT, Koh V, Chiew CJ, Marimuthu K, Thevasagayam NM, Mak TM, et al. Impact of Delta Variant and Vaccination on SARS-CoV-2 Secondary Attack Rate Among Household Close Contacts. Lancet Reg Health West Pac. 2021 Dec;17:100299.

31. Pouwels KB, Pritchard E, Matthews PC, Stoesser N, Eyre DW, Vihta KD, et al. Effect of Delta variant on viral burden and vaccine effectiveness against new SARS-CoV-2 infections in the UK. Nat Med. 2021 Dec;27(12):2127-35.

32. Tartof SY, Slezak JM, Fischer H, Hong V, Ackerson BK, Ranasinghe ON, et al. Effectiveness of mRNA BNT162b2 COVID-19 vaccine up to 6 months in a large integrated health system in the USA: a retrospective cohort study. The Lancet. 2021;398(10309):1407-16.

33. Nunes B, Rodrigues AP, Kislaya I, Cruz C, Peralta-Santos A, Lima J, et al. mRNA vaccine effectiveness against COVID-19-related hospitalisations and deaths in older adults: a cohort study based on data linkage of national health registries in Portugal, February to August 2021. Euro Surveill. 2021 Sep;26(38).

34. Tenforde MW, Self WH, Adams K, Gaglani M, Ginde AA, McNeal T, et al. Association Between mRNA Vaccination and COVID-19 Hospitalization and Disease Severity. JAMA. 2021 Nov 23;326(20):2043-54.

35. Chemaitelly H, Yassine HM, Benslimane FM, Al Khatib HA, Tang P, Hasan MR, et al. mRNA-1273 COVID-19 vaccine effectiveness against the B.1.1.7 and B.1.351 variants and severe COVID-19 disease in Qatar. Nat Med. 2021 Sep;27(9):1614-21.

36. Bruxvoort KJ, Sy LS, Qian L, Ackerson BK, Luo Y, Lee GS, et al. Effectiveness of mRNA-1273 against delta, mu, and other emerging variants of SARS-CoV-2: test negative case-control study. BMJ. 2021 Dec 15;375:e068848.

37. Paris C, Perrin S, Hamonic S, Bourget B, Roue C, Brassard O, et al. Effectiveness of mRNA-BNT162b2, mRNA-1273, and ChAdOx1 nCoV-19 vaccines against COVID-19 in healthcare workers: an observational study using surveillance data. Clin Microbiol Infect. 2021 Nov;27(11):1699 e5- e8.

38. Abu-Raddad LJ, Chemaitelly H, Yassine HM, Benslimane FM, Al Khatib HA, Tang P, et al. Pfizer-BioNTech mRNA BNT162b2 Covid-19 vaccine protection against variants of concern after one versus two doses. J Travel Med. 2021 Oct 11;28(7).

39. Olson SM, Newhams MM, Halasa NB, Price AM, Boom JA, Sahni LC, et al. Effectiveness of Pfizer-BioNTech mRNA Vaccination Against COVID-19 Hospitalization Among Persons Aged 12-18 Years - United States, June-September 2021. MMWR Morb Mortal Wkly Rep. 2021 Oct 22;70(42):1483-8.

40. Lopez Bernal J, Andrews N, Gower C, Robertson C, Stowe J, Tessier E, et al. Effectiveness of the Pfizer-BioNTech and Oxford-AstraZeneca vaccines on covid-19 related symptoms, hospital admissions, and mortality in older adults in England: test negative case-control study. BMJ. 2021 May 13;373:n1088.

41. Grannis SJ, Rowley EA, Ong TC, Stenehjem E, Klein NP, DeSilva MB, et al. Interim Estimates of COVID-19 Vaccine Effectiveness Against COVID-19-Associated Emergency Department or Urgent Care Clinic Encounters and Hospitalizations Among Adults During SARS-CoV-2 B.1.617.2 (Delta) Variant Predominance - Nine States, June-August 2021. MMWR Morb Mortal Wkly Rep. 2021 Sep 17;70(37):1291-3.

42. Grant R, Charmet T, Schaeffer L, Galmiche S, Madec Y, Von Platen C, et al. Impact of SARS-CoV-2 Delta variant on incubation, transmission settings and vaccine effectiveness: Results from a nationwide case-control study in France. Lancet Reg Health Eur. 2022 Feb;13:100278.

43. Rovida F, Cassaniti I, Paolucci S, Percivalle E, Sarasini A, Piralla A, et al. SARS-CoV-2 vaccine breakthrough infections with the alpha variant are asymptomatic or mildly symptomatic among health care workers. Nat Commun. 2021 Oct 15;12(1):6032.

44. Martinez-Baz I, Trobajo-Sanmartin C, Miqueleiz A, Guevara M, Fernandez-Huerta M, Burgui C, et al. Product-specific COVID-19 vaccine effectiveness against secondary infection in close contacts, Navarre, Spain, April to August 2021. Euro Surveill. 2021 Sep;26(39).

45. Haas EJ, Angulo FJ, McLaughlin JM, Anis E, Singer SR, Khan F, et al. Impact and effectiveness of mRNA BNT162b2 vaccine against SARS-CoV-2 infections and COVID-19 cases, hospitalisations, and deaths following a nationwide vaccination campaign in Israel: an observational study using national surveillance data. The Lancet. 2021;397(10287):1819-29.

46. Dagan N, Barda N, Kepten E, Miron O, Perchik S, Katz MA, et al. BNT162b2 mRNA Covid-19 Vaccine in a Nationwide Mass Vaccination Setting. N Engl J Med. 2021 Apr 15;384(15):1412-23.

47. Keegan LT, Truelove S, Lessler J. Analysis of Vaccine Effectiveness Against COVID-19 and the Emergence of Delta and Other Variants of Concern in Utah. JAMA Netw Open. 2021 Dec 1;4(12):e2140906.

48. Kissling E, Hooiveld M, Sandonis Martin V, Martinez-Baz I, William N, Vilcu AM, et al. Vaccine effectiveness against symptomatic SARS-CoV-2 infection in adults aged 65 years and older in primary care: I-MOVE-COVID-19 project, Europe, December 2020 to May 2021. Euro Surveill. 2021 Jul;26(29).

49. Voko Z, Kiss Z, Surjan G, Surjan O, Barcza Z, Palyi B, et al. Nationwide effectiveness of five SARS-CoV-2 vaccines in Hungary-the HUN-VE study. Clin Microbiol Infect. 2022 Mar;28(3):398-404.

50. Carazo S, Talbot D, Boulianne N, Brisson M, Gilca R, Deceuninck G, et al. Single-dose mRNA vaccine effectiveness against SARS-CoV-2 in healthcare workers extending 16 weeks post-vaccination: a test-negative design from Quebec, Canada. Clin Infect Dis. 2021 Aug 30.

51. Skowronski DM, Setayeshgar S, Zou M, Prystajecky N, Tyson JR, Galanis E, et al. Single-dose mRNA Vaccine Effectiveness Against Severe Acute Respiratory Syndrome Coronavirus 2 (SARS-CoV-2), Including Alpha and Gamma Variants: A Test-negative Design in Adults 70 Years and Older in British Columbia, Canada. Clin Infect Dis. 2022 Apr 9;74(7):1158-65.

52. Chemaitelly H, Tang P, Hasan MR, AlMukdad S, Yassine HM, Benslimane FM, et al. Waning of BNT162b2 Vaccine Protection against SARS-CoV-2 Infection in Qatar. N Engl J Med. 2021 Dec 9;385(24):e83.

53. Desai D, Khan AR, Soneja M, Mittal A, Naik S, Kodan P, et al. Effectiveness of an inactivated virus-based SARS-CoV-2 vaccine, BBV152, in India: a test-negative, case-control study. The Lancet Infectious Diseases. 2022;22(3):349-56.

54. Charmet T, Schaeffer L, Grant R, Galmiche S, Cheny O, Von Platen C, et al. Impact of original, B.1.1.7, and B.1.351/P.1 SARS-CoV-2 lineages on vaccine effectiveness of two doses of COVID-19 mRNA vaccines: Results from a nationwide case-control study in France. Lancet Reg Health Eur. 2021 Sep;8:100171.

55. Satwik R, Satwik A, Katoch S, Saluja S. ChAdOx1 nCoV-19 effectiveness during an unprecedented surge in SARS COV-2 infections. Eur J Intern Med. 2021 Nov;93:112-3.

56. Flacco ME, Soldato G, Acuti Martellucci C, Carota R, Di Luzio R, Caponetti A, et al. Interim Estimates of COVID-19 Vaccine Effectiveness in a Mass Vaccination Setting: Data from an Italian Province. Vaccines (Basel). 2021 Jun 10;9(6).

57. Cerqueira-Silva T, Oliveira VA, Boaventura VS, Pescarini JM, Junior JB, Machado TM, et al. Influence of age on the effectiveness and duration of protection of Vaxzevria and CoronaVac vaccines: A population-based study. Lancet Reg Health Am. 2022 Feb;6:100154.

58. Yassi A, Grant JM, Lockhart K, Barker S, Sprague S, Okpani AI, et al. Infection control, occupational and public health measures including mRNA-based vaccination against SARS-CoV-2 infections to protect healthcare workers from variants of concern: A 14-month observational study using surveillance data. PLoS One. 2021;16(7):e0254920.

59. Elliott P, Haw D, Wang H, Eales O, Walters CE, Ainslie KEC, et al. Exponential growth, high prevalence of SARS-CoV-2, and vaccine effectiveness associated with the Delta variant. Science. 2021 Dec 17;374(6574):eabl9551.

60. Veneti L, Valcarcel Salamanca B, Seppala E, Starrfelt J, Storm ML, Bragstad K, et al. No difference in risk of hospitalization between reported cases of the SARS-CoV-2 Delta variant and Alpha variant in Norway. Int J Infect Dis. 2022 Feb;115:178-84.

61. Grgic Vitek M, Klavs I, Ucakar V, Serdt M, Mrzel M, Vrh M, et al. Vaccine effectiveness against severe acute respiratory infections (SARI) COVID-19 hospitalisations estimated from real-world surveillance data, Slovenia, October 2021. Euro Surveill. 2022 Jan;27(1).

62. Li XN, Huang Y, Wang W, Jing QL, Zhang CH, Qin PZ, et al. Effectiveness of inactivated SARS-CoV-2 vaccines against the Delta variant infection in Guangzhou: a test-negative case-control real-world study. Emerg Microbes Infect. 2021 Dec;10(1):1751-9.

63. Chin ET, Leidner D, Zhang Y, Long E, Prince L, Li Y, et al. Effectiveness of the mRNA-1273 Vaccine during a SARS-CoV-2 Delta Outbreak in a Prison. N Engl J Med. 2021 Dec 9;385(24):2300-1.

64. Rosenberg ES, Dorabawila V, Easton D, Bauer UE, Kumar J, Hoen R, et al. Covid-19 Vaccine Effectiveness in New York State. N Engl J Med. 2022 Jan 13;386(2):116-27.

65. Tenforde MW, Patel MM, Ginde AA, Douin DJ, Talbot HK, Casey JD, et al. Effectiveness of Severe Acute Respiratory Syndrome Coronavirus 2 Messenger RNA Vaccines for Preventing Coronavirus Disease 2019 Hospitalizations in the United States. Clinical Infectious Diseases. 2021;74(9):1515-24.

66. Baum U, Poukka E, Palmu AA, Salo H, Lehtonen TO, Leino T. Effectiveness of vaccination against SARS-CoV-2 infection and Covid-19 hospitalisation among Finnish elderly and chronically ill-An interim analysis of a nationwide cohort study. PLoS One. 2021;16(11):e0258704.

67. Sheikh A, McMenamin J, Taylor B, Robertson C. SARS-CoV-2 Delta VOC in Scotland: demographics, risk of hospital admission, and vaccine effectiveness. The Lancet. 2021;397(10293):2461-2.

68. Hall VJ, Foulkes S, Saei A, Andrews N, Oguti B, Charlett A, et al. COVID-19 vaccine coverage in health-care workers in England and effectiveness of BNT162b2 mRNA vaccine against infection (SIREN): a prospective, multicentre, cohort study. The Lancet. 2021;397(10286):1725-35.

69. de Gier B, Andeweg S, Backer JA, surveillance RC-, epidemiology t, Hahne SJ, et al. Vaccine effectiveness against SARS-CoV-2 transmission to household contacts during dominance of Delta variant (B.1.617.2), the Netherlands, August to September 2021. Euro Surveill. 2021 Nov;26(44).

70. Bhattacharya A, Ranjan P, Ghosh T, Agarwal H, Seth S, Maher GT, et al. Evaluation of the dose-effect association between the number of doses and duration since the last dose of COVID-19 vaccine, and its efficacy in preventing the disease and reducing disease severity: A single centre, cross-sectional analytical study from India. Diabetes Metab Syndr. 2021 Sep-Oct;15(5):102238.

71. Nanduri S, Pilishvili T, Derado G, Soe MM, Dollard P, Wu H, et al. Effectiveness of Pfizer-BioNTech and Moderna Vaccines in Preventing SARS-CoV-2 Infection Among Nursing Home Residents Before and During Widespread Circulation of the SARS-CoV-2 B.1.617.2 (Delta) Variant - National Healthcare Safety Network, March 1-August 1, 2021. MMWR Morb Mortal Wkly Rep. 2021 Aug 27;70(34):1163-6.

72. Tenforde MW, Self WH, Naioti EA, Ginde AA, Douin DJ, Olson SM, et al. Sustained Effectiveness of Pfizer-BioNTech and Moderna Vaccines Against COVID-19 Associated Hospitalizations Among Adults - United States, March-July 2021. MMWR Morb Mortal Wkly Rep. 2021 Aug 27;70(34):1156-62.

73. Katikireddi SV, Cerqueira-Silva T, Vasileiou E, Robertson C, Amele S, Pan J, et al. Two-dose ChAdOx1 nCoV-19 vaccine protection against COVID-19 hospital admissions and deaths over time: a retrospective, population-based cohort study in Scotland and Brazil. The Lancet. 2022;399(10319):25-35.

74. Shrotri M, Krutikov M, Palmer T, Giddings R, Azmi B, Subbarao S, et al. Vaccine effectiveness of the first dose of ChAdOx1 nCoV-19 and BNT162b2 against SARS-CoV-2 infection in residents of long-term care facilities in England (VIVALDI): a prospective cohort study. The Lancet Infectious Diseases. 2021;21(11):1529-38.

75. Hyams C, Marlow R, Maseko Z, King J, Ward L, Fox K, et al. Effectiveness of BNT162b2 and ChAdOx1 nCoV-19 COVID-19 vaccination at preventing hospitalisations in people aged at least 80 years: a test-negative, case-control study. The Lancet Infectious Diseases. 2021;21(11):1539-48.

76. Hitchings MDT, Ranzani OT, Torres MSS, de Oliveira SB, Almiron M, Said R, et al. Effectiveness of CoronaVac among healthcare workers in the setting of high SARS-CoV-2 Gamma variant transmission in Manaus, Brazil: A test-negative case-control study. Lancet Reg Health Am. 2021 Sep;1:100025.

77. Accorsi EK, Britton A, Fleming-Dutra KE, Smith ZR, Shang N, Derado G, et al. Association Between 3 Doses of mRNA COVID-19 Vaccine and Symptomatic Infection Caused by the SARS-CoV-2 Omicron and Delta Variants. Jama. 2022;327(7).

78. Fabiani M, Puopolo M, Filia A, Sacco C, Mateo-Urdiales A, Spila Alegiani S, et al. Effectiveness of an mRNA vaccine booster dose against SARS-CoV-2 infection and severe COVID-19 in persons aged >/=60 years and other high-risk groups during predominant circulation of the delta variant in Italy, 19 July to 12 December 2021. Expert Rev Vaccines. 2022 Apr 15:1-8.

79. June Choe Y, Yi S, Hwang I, Kim J, Park YJ, Cho E, et al. Safety and effectiveness of BNT162b2 mRNA Covid-19 vaccine in adolescents. Vaccine. 2022 Jan 31;40(5):691-4.

80. Britton A, Fleming-Dutra KE, Shang N, Smith ZR, Dorji T, Derado G, et al. Association of COVID-19 Vaccination With Symptomatic SARS-CoV-2 Infection by Time Since Vaccination and Delta Variant Predominance. JAMA. 2022 Mar 15;327(11):1032-41.

81. Fabiani M, Puopolo M, Morciano C, Spuri M, Spila Alegiani S, Filia A, et al. Effectiveness of mRNA vaccines and waning of protection against SARS-CoV-2 infection and severe covid-19 during predominant circulation of the delta variant in Italy: retrospective cohort study. BMJ. 2022 Feb 10;376:e069052.

82. Mingshuang L, Qianqian L, Dan W, Lin T, Xiaoqi W, Tingting Y, et al. Association of COVID-19 Vaccination and Clinical Severity of Patients Infected with Delta or Omicron Variants — China, May 21, 2021–February 28, 2022. China CDC Weekly. 2022;4(14):293-7.

83. Kong TUoH. HKUMed proposes forward planning after Hong Kong’s fifth wave of Omicron BA.2; 2022.

84. Ferdinands JM, Rao S, Dixon BE, Mitchell PK, DeSilva MB, Irving SA, et al. Waning 2-Dose and 3-Dose Effectiveness of mRNA Vaccines Against COVID-19-Associated Emergency Department and Urgent Care Encounters and Hospitalizations Among Adults During Periods of Delta and Omicron Variant Predominance - VISION Network, 10 States, August 2021-January 2022. MMWR Morb Mortal Wkly Rep. 2022 Feb 18;71(7):255-63.

85. Nyberg T, Ferguson NM, Nash SG, Webster HH, Flaxman S, Andrews N, et al. Comparative analysis of the risks of hospitalisation and death associated with SARS-CoV-2 omicron (B.1.1.529) and delta (B.1.617.2) variants in England: a cohort study. The Lancet. 2022;399(10332):1303-12.

86. McLean HQ, McClure DL, King JP, Meece JK, Pattinson D, Neumann G, et al. mRNA COVID-19 vaccine effectiveness against SARS-CoV-2 infection in a prospective community cohort, rural Wisconsin, November 2020 to December 2021. Influenza Other Respir Viruses. 2022 Feb 18.

87. Chung JR, Kim SS, Belongia EA, McLean HQ, King JP, Nowalk MP, et al. Vaccine effectiveness against COVID-19 among symptomatic persons aged >/=12 years with reported contact with COVID-19 cases, February-September 2021. Influenza Other Respir Viruses. 2022 Feb 15.

88. Pramod S, Govindan D, Ramasubramani P, Kar SS, Aggarwal R, group Jves. Effectiveness of Covishield vaccine in preventing Covid-19 - A test-negative case-control study. Vaccine. 2022 May 26;40(24):3294-7.

89. Tseng HF, Ackerson BK, Luo Y, Sy LS, Talarico CA, Tian Y, et al. Effectiveness of mRNA-1273 against SARS-CoV-2 Omicron and Delta variants. Nat Med. 2022 May;28(5):1063-71.

90. McKeigue PM, McAllister DA, Hutchinson SJ, Robertson C, Stockton D, Colhoun HM. Vaccine efficacy against severe COVID-19 in relation to delta variant (B.1.617.2) and time since second dose in patients in Scotland (REACT-SCOT): a case-control study. The Lancet Respiratory Medicine. 2022;10(6):566-72.

91. Hu Z, Tao B, Li Z, Song Y, Yi C, Li J, et al. Effectiveness of inactivated COVID-19 vaccines against severe illness in B.1.617.2 (Delta) variant-infected patients in Jiangsu, China. Int J Infect Dis. 2022 Mar;116:204-9.

92. Polinski JM, Weckstein AR, Batech M, Kabelac C, Kamath T, Harvey R, et al. Durability of the Single-Dose Ad26.COV2.S Vaccine in the Prevention of COVID-19 Infections and Hospitalizations in the US Before and During the Delta Variant Surge. JAMA Netw Open. 2022 Mar 1;5(3):e222959.

93. Kretzschmar MEE, Gram MA, Nielsen J, Schelde AB, Nielsen KF, Moustsen-Helms IR, et al. Vaccine effectiveness against SARS-CoV-2 infection, hospitalization, and death when combining a first dose ChAdOx1 vaccine with a subsequent mRNA vaccine in Denmark: A nationwide population-based cohort study. PLOS Medicine. 2021;18(12).

94. Robles-Fontán MM, Nieves EG, Cardona-Gerena I, Irizarry RA. Effectiveness estimates of three COVID-19 vaccines based on observational data from Puerto Rico. The Lancet Regional Health - Americas. 2022;9.

95. Tartof SY, Slezak JM, Puzniak L, Hong V, Xie F, Ackerson BK, et al. Durability of BNT162b2 vaccine against hospital and emergency department admissions due to the omicron and delta variants in a large health system in the USA: a test-negative case–control study. The Lancet Respiratory Medicine. 2022.

96. Klein NP, Stockwell MS, Demarco M, Gaglani M, Kharbanda AB, Irving SA, et al. Effectiveness of COVID-19 Pfizer-BioNTech BNT162b2 mRNA Vaccination in Preventing COVID-19-Associated Emergency Department and Urgent Care Encounters and Hospitalizations Among Nonimmunocompromised Children and Adolescents Aged 5-17 Years - VISION Network, 10 States, April 2021-January 2022. MMWR Morb Mortal Wkly Rep. 2022 Mar 4;71(9):352-8.

97. Price AM, Olson SM, Newhams MM, Halasa NB, Boom JA, Sahni LC, et al. BNT162b2 Protection against the Omicron Variant in Children and Adolescents. N Engl J Med. 2022 May 19;386(20):1899-909.

98. Jara A, Undurraga EA, Zubizarreta JR, González C, Pizarro A, Acevedo J, et al. Effectiveness of homologous and heterologous booster doses for an inactivated SARS-CoV-2 vaccine: a large-scale prospective cohort study. The Lancet Global Health. 2022;10(6):e798-e806.

99. Tenforde MW, Self WH, Gaglani M, Ginde AA, Douin DJ, Talbot HK, et al. Effectiveness of mRNA Vaccination in Preventing COVID-19-Associated Invasive Mechanical Ventilation and Death - United States, March 2021-January 2022. MMWR Morb Mortal Wkly Rep. 2022 Mar 25;71(12):459-65.

100. Natarajan K, Prasad N, Dascomb K, Irving SA, Yang DH, Gaglani M, et al. Effectiveness of Homologous and Heterologous COVID-19 Booster Doses Following 1 Ad.26.COV2.S (Janssen [Johnson & Johnson]) Vaccine Dose Against COVID-19-Associated Emergency Department and Urgent Care Encounters and Hospitalizations Among Adults - VISION Network, 10 States, December 2021-March 2022. MMWR Morb Mortal Wkly Rep. 2022 Apr 1;71(13):495-502.

101. Suarez Castillo M, Khaoua H, Courtejoie N. Vaccine-induced and naturally-acquired protection against Omicron and Delta symptomatic infection and severe COVID-19 outcomes, France, December 2021 to January 2022. Euro Surveill. 2022 Apr;27(16).

102. Fowlkes AL, Yoon SK, Lutrick K, Gwynn L, Burns J, Grant L, et al. Effectiveness of 2-Dose BNT162b2 (Pfizer BioNTech) mRNA Vaccine in Preventing SARS-CoV-2 Infection Among Children Aged 5-11 Years and Adolescents Aged 12-15 Years - PROTECT Cohort, July 2021-February 2022. MMWR Morb Mortal Wkly Rep. 2022 Mar 18;71(11):422-8.

103. Sharma A, Oda G, Holodniy M. Effectiveness of mRNA-based vaccines during the emergence of SARS-CoV-2 Omicron variant. Clin Infect Dis. 2022 Apr 27.

104. Andrews N, Stowe J, Kirsebom F, Toffa S, Rickeard T, Gallagher E, et al. Covid-19 Vaccine Effectiveness against the Omicron (B.1.1.529) Variant. N Engl J Med. 2022 Apr 21;386(16):1532-46.

105. Lauring AS, Tenforde MW, Chappell JD, Gaglani M, Ginde AA, McNeal T, et al. Clinical severity of, and effectiveness of mRNA vaccines against, covid-19 from omicron, delta, and alpha SARS-CoV-2 variants in the United States: prospective observational study. BMJ. 2022 Mar 9;376:e069761.

106. Thompson MG, Natarajan K, Irving SA, Rowley EA, Griggs EP, Gaglani M, et al. Effectiveness of a Third Dose of mRNA Vaccines Against COVID-19-Associated Emergency Department and Urgent Care Encounters and Hospitalizations Among Adults During Periods of Delta and Omicron Variant Predominance - VISION Network, 10 States, August 2021-January 2022. MMWR Morb Mortal Wkly Rep. 2022 Jan 21;71(4):139-45.

107. Sritipsukho P, Khawcharoenporn T, Siribumrungwong B, Damronglerd P, Suwantarat N, Satdhabudha A, et al. Comparing real-life effectiveness of various COVID-19 vaccine regimens during the delta variant-dominant pandemic: a test-negative case-control study. Emerg Microbes Infect. 2022 Dec;11(1):585-92.

108. Accorsi EK, Britton A, Shang N, Fleming-Dutra KE, Link-Gelles R, Smith ZR, et al. Effectiveness of Homologous and Heterologous Covid-19 Boosters against Omicron. N Engl J Med. 2022 Jun 23;386(25):2433-5.

109. Cerqueira-Silva T, de Araujo Oliveira V, Paixao ES, Junior JB, Penna GO, Werneck GL, et al. Duration of protection of CoronaVac plus heterologous BNT162b2 booster in the Omicron period in Brazil. Nat Commun. 2022 Jul 18;13(1):4154.

110. Richterman A, Behrman A, Brennan PJ, O'Donnell JA, Snider CK, Chaiyachati KH. Durability of SARS-CoV-2 mRNA Booster Vaccine Protection Against Omicron Among Health Care Workers with a Vaccine Mandate. Clin Infect Dis. 2022 Jun 6.

111. Chemaitelly H, Ayoub HH, AlMukdad S, Coyle P, Tang P, Yassine HM, et al. Duration of mRNA vaccine protection against SARS-CoV-2 Omicron BA.1 and BA.2 subvariants in Qatar. Nat Commun. 2022 Jun 2;13(1):3082.

112. Altarawneh HN, Chemaitelly H, Ayoub HH, Tang P, Hasan MR, Yassine HM, et al. Effects of Previous Infection and Vaccination on Symptomatic Omicron Infections. N Engl J Med. 2022 Jul 7;387(1):21-34.

113. Kirsebom FCM, Andrews N, Stowe J, Toffa S, Sachdeva R, Gallagher E, et al. COVID-19 vaccine effectiveness against the omicron (BA.2) variant in England. The Lancet Infectious Diseases. 2022;22(7):931-3.

114. McMenamin ME, Nealon J, Lin Y, Wong JY, Cheung JK, Lau EHY, et al. Vaccine effectiveness of one, two, and three doses of BNT162b2 and CoronaVac against COVID-19 in Hong Kong: a population-based observational study. The Lancet Infectious Diseases. 2022.

Table S3. Search strategy for peer-reviewed databases.

| **Database** | **Search strategy** | **Records** |
| --- | --- | --- |
| PubMed | (COVID-19[Mesh] OR SARS-CoV-2[Mesh] OR "COVID-19 Vaccines"[Mesh] OR COVID-19[Title/Abstract] OR SARS-CoV-2[Title/Abstract])  AND  (vaccines[Mesh] OR vaccination[Mesh] OR immunization[Mesh] OR vaccin*[Title/Abstract] OR immuniz*[Title/Abstract] OR effectiveness[Title/Abstract] OR eff* [Title/Abstract])  AND  (variant*[Title/Abstract] OR VOC[Title/Abstract] OR B.1.1.7[Title/Abstract] OR B.1.351[Title/Abstract] OR P.1[Title/Abstract] OR B.1.617.2[Title/Abstract] OR B.1.1.529[Title/Abstract] OR alpha[Title/Abstract] OR beta[Title/Abstract] OR gamma[Title/Abstract] OR delta[Title/Abstract] OR omicron[Title/Abstract])  Search limits: 1 December 2020 to 5 Aug 2022 | 6869 |
| Embase | ('coronavirus disease 2019'/exp OR 'Severe acute respiratory syndrome coronavirus 2'/exp OR (COVID-19 OR SARS-CoV-2):ti,ab)  AND  (vaccine/exp OR vaccination/exp OR immunization/exp OR effectiveness/exp OR (vaccin* OR immuniz* OR eff*):ti,ab)  AND  ((variant* OR VOC OR B.1.1.7 OR B.1.351 OR P.1 OR B.1.617.2 OR B.1.1.529 OR alpha OR beta OR gamma OR delta OR omicron):ti,ab)  Search limits: 1 December 2020 to 5 Aug 2022 | 4436 |
| Cochrane Library | ([mh vaccines] OR [mh SARS-CoV-2] OR COVID-19:ti,ab OR SARS-CoV-2:ti,ab)  AND  ([mh vaccines] OR [mh vaccination] OR [mh immunization] OR [mh effectiveness] OR (vaccin*:ti,ab) OR immuniz*:ti,ab OR eff*:ti,ab)  AND  ((variant* OR VOC OR B.1.1.7 OR B.1.351 OR P.1 OR B.1.617.2 OR B.1.1.529 OR alpha OR beta OR gamma OR delta OR omicron):ti,ab)  Search limits: 1 December 2020 to 5 Aug 2022 | 97 |

Table S4. Interpretation of clinical outcomes of interest.

| **Clinical outcomes** | **Interpretation** |
| --- | --- |
| SARS-Cov-2 infection | Laboratory-confirmed SARS-Cov-2 infection through polymerase chain reaction or antigen test. |
| COVID-19 related hospitalization | Any inpatient hospital admission with a positive, laboratory confirmed COVID-19. |
| COVID-19 associated emergency department or urgent care (ED or UC) visits | An emergency department or urgent care clinic visit associated with laboratory-confirmed SARS-CoV-2 infection and a diagnosis consistent with COVID19–like illness, which was defined as a clinical diagnosis of acute respiratory illness (e.g., Covid-19, respiratory failure, or pneumonia) or signs or symptoms (e.g., cough, fever, dyspnea, vomiting, or diarrhea) |
| Severe, critical or fatal COVID-19 disease | At least one of the following: the need for oxygen supplementation, admission to intensive care, mechanical ventilation, or death |
| COVID-19 related intensive care unit (ICU) admission | Any inpatient admission to intensive care unit with a positive, laboratory confirmed COVID-19 |
| COVID-19 related death | Occurrence of any death with a positive, laboratory confirmed COVID-19 |

Table S5. VE of full and partial vaccination against Alpha variant.

| **Clinical outcomes** | **Study (n)** | **Vaccine Effectiveness** | ***I* ^2^** |
| --- | --- | --- | --- |
| **Full vaccination** |  |  |  |
| **Confirmed infection** |  |  |  |
| **Summary** | **33** | 86.8 (82.9 to 89.7) | 98.7 |
| **By vaccine product** |  |  |  |
| mRNA vaccine | 32 | 88.3 (84.8 to 91.0) | 98.2 |
| BNT162b2 | 23 | 87.5 (82.7 to 91.0) | 98.7 |
| mRNA-1273 | 4 | 94.5 (89.5 to 97.1) | 91.8 |
| ChAdOx1 nCoV-19 | 8 | 73.6 (67.9 to 78.3) | 87.8 |
| **By study population** |  |  |  |
| General population | 18 | 89.6 (85.1 to 92.8) | 99.3 |
| The older | 10 | 84.5 (78.2 to 89.0) | 99.0 |
| Health worker | 10 | 86.6 (79.2 to 91.3) | 71.9 |
| The close contacts of COVID-19 case | 3 | 67.0 (62.2 to 71.2) | 67.1 |
| **COVID-19 related hospitalization** |  |  |  |
| **Summary** | **11** | 90.4 (83.8 to 94.3) | 97.4 |
| **By vaccine product** |  |  |  |
| mRNA vaccine | 11 | 91.6 (85.8 to 95.1) | 97.3 |
| BNT162b2 | 5 | 95.5 (87.9 to 98.3) | 98.1 |
| By study population |  |  |  |
| General population | 8 | 92.1 (82.9 to 96.4) | 97.9 |
| The older | 4 | 95.4 (87.1 to 98.4) | 96.2 |
| **COVID-19 related ICU admission ¶§** |  |  |  |
| **Summary** | **3** | 96.0 (89.9 to 98.4) | 97.8 |
| **Severe, critical or fatal COVID-19 disease ¶** |  |  |  |
| **Summary** | **3** | 92.2 (88.0 to 94.9) | 83.3 |
| **By study population** |  |  |  |
| General population | 3 | 91.1 (85.0 to 94.7) | 85.2 |
| **COVID-19 related death** |  |  |  |
| **Summary** | **5** | 94.2 (85.5 to 97.7) | 98.0 |
| **By vaccine product** |  |  |  |
| mRNA vaccine | 5 | 94.3 (89.1 to 97.0) | 97.1 |
| BNT162b2 | 4 | 95.9 (90.3 to 98.2) | 98.0 |
| **By study population** |  |  |  |
| General population | 4 | 95.7 (87.7 to 98.5) | 98.4 |
| The older | 4 | 93.3 (87.2 to 96.5) | 95.9 |
|  |  |  |  |
| **Partial vaccination** |  |  |  |
| **Confirmed infection** |  |  |  |
| **Summary** | **27** | 46.1 (34.2 to 55.8) | 95.8 |
| **COVID-19 related hospitalization** |  |  |  |
| **Summary** | **10** | 69.9 (59.2 to 77.7) | 93.0 |
| **Severe, critical or fatal COVID-19 disease** |  |  |  |
| **Summary** | **3** | 68.6 (59.0 to 75.9) | 89.3 |
| **COVID-19 related death** |  |  |  |
| **Summary** | **5** | 66.8 (52.7 to 76.7) | 78.6 |

¶Studies that estimated VE all used mRNA vaccines. §All studies conducted in general population.

Table S6. VE of full and partial vaccination against Beta variant.

| **Clinical outcome** | **Study (n)** | **Vaccine Effectiveness** | ***I* ^2^** |
| --- | --- | --- | --- |
| **Full vaccination** |  |  |  |
| **Confirmed infection** |  |  |  |
| **Summary** | **5** | 72.8 (65.0 to 78.9) | 83.8 |
| **By vaccine product** |  |  |  |
| BNT162b2 | 4 | 70.6 (67.7 to 73.2) | 62.9 |
| **By study population** |  |  |  |
| General population | 3 | 75.2 (67.7 to 81.0) | 88.4 |
|  |  |  |  |
| **Partial vaccination** |  |  |  |
| **Confirmed infection** |  |  |  |
| **Summary** | **5** | 47.9 (35.0 to 58.3) | 83.5 |

Table S7. VE of full and partial vaccination against Gamma variant.

| **Clinical outcome** | **Study (n)** | **Vaccine Effectiveness** | ***I* ^2^** |
| --- | --- | --- | --- |
| **Full vaccination** |  |  |  |
| **Confirmed infection** |  |  |  |
| **Summary** | 7 | 71.9 (64.6 to 77.6) | 99.4 |
| **By vaccine product** |  |  |  |
| ChAdOx1 nCoV-19 | 3 | 73.4 (65.9 to 79.2) | 98.3 |
| CoronaVac | 3 | 53.1 (52.2 to 53.9) | 35.9 |
| **By study population** |  |  |  |
| General population | 3 | 73.2 (63.8 to 80.2) | 99.7 |
| The older | 3 | 67.2 (59.8 to 73.2) | 97.3 |
| **COVID-19 related hospitalization** |  |  |  |
| **Summary** | 4 | 78.4 (70.1 to 84.4) | 98.7 |
| **By vaccine product** |  |  |  |
| ChAdOx1 nCoV-19 | 3 | 85.5 (75.6 to 91.4) | 95.8 |
| **By study population** |  |  |  |
| The older | 3 | 76.6 (64.9 to 84.4) | 96.9 |
| **COVID-19 related death** |  |  |  |
| **Summary** | 3 | 82.2 (74.1 to 87.8) | 97.9 |
| **By study population** |  |  |  |
| The older | 3 | 82.0 (70.5 to 89.1) | 96.1 |
|  |  |  |  |
| **Partial vaccination** |  |  |  |
| **Confirmed infection** |  |  |  |
| **Summary** | 8 | 38.0 (33.8 to 41.9) | 99.2 |
| **COVID-19 related hospitalization** |  |  |  |
| **Summary** | 4 | 51.3 (39.3 to 60.9) | 99.4 |
| **COVID-19 related death** |  |  |  |
| **Summary** | 3 | 52.9 (33.1 to 66.9) | 98.7 |

Table S8. VE of booster, full and partial vaccination against Delta variant.

| **Clinical outcomes** | **Study (n)** | **Vaccine Effectiveness** | ***I* ^2^** |
| --- | --- | --- | --- |
| **Booster vaccination** |  |  |  |
| **Confirmed infection §** |  |  |  |
| **Summary** | 7 | 93.3 (91.7 to 94.6) | 99.0 |
| **By vaccine product** |  |  |  |
| mRNA vaccine | 6 | 93.6 (92.1 to 94.8) | 96.2 |
| **COVID-19 related hospitalization §** |  |  |  |
| **Summary** | 11 | 92.8 (89.1 to 95.2) | 98.4 |
| **By vaccine product** |  |  |  |
| mRNA vaccine | 7 | 91.9 (88.2 to 94.5) | 97.0 |
| **COVID-19 associated ED and UC ¶§** |  |  |  |
| **Summary** | 3 | 92.7 (88.7 to 95.3) | 98.7 |
| **Severe, critical or fatal COVID-19 disease** |  |  |  |
| **Summary** | 3 | 93.8 (91.7 to 95.3) | 0.0 |
|  |  |  |  |
| **Full vaccination** |  |  |  |
| **Confirmed infection** |  |  |  |
| **Summary** | 43 | 70.9 (68.9 to 72.7) | 98.1 |
| **By vaccine product** |  |  |  |
| mRNA vaccine | 31 | 77.8 (74.2 to 80.9) | 99.6 |
| BNT162b2 | 20 | 79.3 (76.7 to 81.5) | 98.6 |
| mRNA-1273 | 12 | 78.5 (75.0 to 81.5) | 99.7 |
| ChAdOx1 nCoV-19 | 10 | 62.6 (60.9 to 64.2) | 83.9 |
| Ad26.COV2.S | 5 | 63.9 (54.5 to 71.4) | 92.7 |
| **By study population** |  |  |  |
| General population | 23 | 73.3 (69.1 to 76.9) | 98.5 |
| The older | 8 | 64.0 (54.8 to 71.4) | 99.0 |
| Health worker | 7 | 50.9 (44.8 to 56.3) | 74.7 |
| The close contacts of COVID-19 case | 5 | 61.5 (50.2 to 70.2) | 64.0 |
| Adolescent | 6 | 90.4 (80.6 to 95.2) | 97.4 |
| **COVID-19 related hospitalization** |  |  |  |
| **Summary** | 24 | 84.9 (82.4 to 87.1) | 97.9 |
| **By vaccine product** |  |  |  |
| mRNA vaccine | 17 | 88.5 (84.9 to 91.2) | 97.9 |
| BNT162b2 | 8 | 88.9 (81.8 to 93.3) | 98.7 |
| mRNA-1273 | 3 | 91.2 (90.1 to 92.2) | 79.4 |
| ChAdOx1 nCoV-19 | 6 | 80.7 (68.4 to 88.2) | 99.1 |
| **By study population** |  |  |  |
| General population | 19 | 85.4 (82.1 to 88.0) | 98.3 |
| The older | 6 | 82.6 (76.7 to 87.1) | 93.1 |
| **COVID-19 associated ED and UC** |  |  |  |
| **Summary** | 5 | 78.5 (70.4 to 84.4) | 95.7 |
| **By vaccine product** |  |  |  |
| mRNA vaccine | 4 | 77.5 (66.5 to 85.0) | 96.3 |
| **By study population** |  |  |  |
| General population | 4 | 76.8 (67.5 to 83.4) | 96.7 |
| **COVID-19 related ICU admission** |  |  |  |
| **Summary** | 5 | 88.2 (85.2 to 90.6) | 62.9 |
| **By vaccine product** |  |  |  |
| ChAdOx1 nCoV-19 | 3 | 89.5 (85.0 to 92.6) | 52.1 |
| **By study population** |  |  |  |
| General population | 3 | 88.8 (85.8 to 91.2) | 71.6 |
| **Severe, critical or fatal COVID-19 disease** |  |  |  |
| **Summary** | 6 | 88.8 (81.1 to 93.3) | 92.4 |
| **By vaccine product** |  |  |  |
| mRNA vaccine | 5 | 90.5 (84.9 to 94.0) | 80.3 |
| **By study population** |  |  |  |
| General population | 4 | 87.9 (79.0 to 93.1) | 94.6 |
| The older | 4 | 79.6 (67.2 to 87.3) | 94.4 |
| **COVID-19 related death** |  |  |  |
| **Summary** | 4 | 90.3 (82.4 to 94.7) | 92.0 |
| **By vaccine product** |  |  |  |
| ChAdOx1 nCoV-19 | 3 | 92.9 (91.4 to 94.1) | 3.3 |
| **By study population** |  |  |  |
| General population | 3 | 89.5 (80.5 to 94.3) | 93.8 |
|  |  |  |  |
| **Partial vaccination** |  |  |  |
| **Confirmed infection** |  |  |  |
| **Summary** | 25 | 50.0 (46.9 to 52.9) | 97.3 |
| **COVID-19 related hospitalization** |  |  |  |
| **Summary** | 10 | 68.3 (51.2 to 79.4) | 97.7 |
| **Severe, critical or fatal COVID-19 disease** |  |  |  |
| **Summary** | 4 | 69.4 (52.3 to 80.3) | 85.3 |
| **COVID-19 related death** |  |  |  |
| **Summary** | 3 | 89.2 (84.2 to 92.6) | 23.9 |

Table S9. VE of booster, full and partial vaccination against Omicron variant.

| **Clinical outcomes** | **Study (n)** | **Vaccine Effectiveness** | ***I* ^2^** |
| --- | --- | --- | --- |
| **Booster vaccination** |  |  |  |
| **Confirmed infection** |  |  |  |
| **Summary** | 12 | 57.6 (55.1 to 59.9) | 96.1 |
| **By vaccine product** |  |  |  |
| mRNA vaccine | 10 | 60.8 (58.6 to 62.9) | 95.7 |
| BNT162b2 | 7 | 57.0 (51.1 to 62.2) | 95.9 |
| mRNA-1273 | 6 | 63.1 (55.6 to 69.4) | 92.6 |
| **By study population** |  |  |  |
| General population | 11 | 57.5 (53.8 to 61.0) | 96.4 |
| **By booster regimen** † |  |  |  |
| Homologous booster vaccination | 10 | 57.0 (52.3 to 61.3) | 97.2 |
| Homologous booster with adenovirus or inactivated vaccine | 3 | 41.9 (24.1 to 55.6) | 91.4 |
| Heterologous booster vaccination | 4 | 58.2 (52.1 to 63.5) | 76.4 |
| Heterologous booster adenovirus or inactivated vaccine | 3 | 54.8 (50.6 to 58.6) | 65.8 |
| **By sublineage** |  |  |  |
| BA.1 | 4 | 55.8 (51.0 to 60.0) | 0.0 |
| BA.2 | 4 | 53.2 (45.1 to 60.2) | 91.6 |
| **COVID-19 related hospitalization §** |  |  |  |
| **Summary** | 10 | 83.4 (80.7 to 85.8) | 91.6 |
| **By vaccine product** |  |  |  |
| mRNA vaccine | 9 | 85.5 (82.5 to 88.0) | 88.4 |
| BNT162b2 | 3 | 80.5 (77.6 to 83.0) | 41.1 |
| **COVID-19-Associated ED and UC §** |  |  |  |
| **Summary** | 5 | 78.1 (71.2 to 83.4) | 93.7 |
| **By vaccine product** |  |  |  |
| mRNA vaccine | 5 | 81.3 (78.1 to 84.0) | 76.9 |
| **Severe, critical or fatal COVID-19 disease** |  |  |  |
| **Summary** | 6 | 95.5 (92.0 to 97.5) | 94.9 |
| **By vaccine product** |  |  |  |
| mRNA vaccine | 4 | 96.4 (92.3 to 98.3) | 85.0 |
| **By study population** |  |  |  |
| General population | 5 | 95.7 (89.4 to 98.3) | 95.8 |
| **COVID-19 related death §** |  |  |  |
| **Summary** | 3 | 94.9 (89.2 to 97.6) | 96.0 |
| **By vaccine product** |  |  |  |
| mRNA vaccine | 3 | 93.5 (86.7 to 96.8) | 95.4 |
|  |  |  |  |
| **Full vaccination** |  |  |  |
| **Confirmed Infection** |  |  |  |
| **Summary** | 12 | 23.5 (17.0 to 29.5) | 94.8 |
| **By vaccine product** |  |  |  |
| mRNA vaccine | 9 | 25.8 (16.8 to 33.8) | 93.6 |
| BNT162b2 | 7 | 27.1 (13.4 to 38.6) | 92.7 |
| mRNA-1273 | 5 | 24.9 (4.9 to 40.8) | 96.0 |
| **By study population** |  |  |  |
| General population | 10 | 22.7 (15.2 to 29.7) | 95.8 |
| **COVID-19 related hospitalization §** |  |  |  |
| **Summary** | 12 | 56.5 (50.9 to 61.4) | 84.8 |
| **By vaccine product** |  |  |  |
| mRNA vaccine | 9 | 60.6 (55.9 to 64.7) | 67.0 |
| BNT162b2 | 5 | 58.8 (50.4 to 65.8) | 68.7 |
| **COVID-19 Associated ED and UC** |  |  |  |
| **Summary** | 5 | 36.1 (25.6 to 45.2) | 93.6 |
| **By vaccine product** |  |  |  |
| mRNA vaccine | 4 | 36.8 (24.5 to 47.1) | 86.0 |
| By study population |  |  |  |
| General population | 4 | 38.9 (31.8 to 45.3) | 93.5 |
| **Severe, critical or fatal COVID-19 disease** |  |  |  |
| **Summary** | 7 | 77.6 (66.6 to 85.0) | 85.6 |
| **By vaccine product** |  |  |  |
| mRNA vaccine | 5 | 80.6 (70.6 to 87.2) | 75.8 |
| **By study population** |  |  |  |
| General population | 5 | 78.0 (65.6 to 85.9) | 89.3 |
| **COVID-19 related death §** |  |  |  |
| **Summary** | 3 | 82.4 (66.1 to 90.9) | 93.5 |
| **By vaccine product** |  |  |  |
| mRNA vaccine | 3 | 72.9 (54.2 to 83.9) | 94.8 |
|  |  |  |  |
| **Partial vaccination** |  |  |  |
| **Confirmed infection** |  |  |  |
| **Summary** | 6 | 25.9 (20.0 to 34.9) | 97.4 |

†Five COVID-19 vaccines were combined into six booster regimens including two dose ChAdOx1 nCoV-19 with BNT162b2 or mRNA-1273 booster, two dose BNT162b2 with mRNA-1273 booster, two dose mRNA-1273 with BNT162b2 booster, one dose Ad26.COV2-S with BNT162b2 or mRNA-1273 booster and two dose CoronaVac with BNT162b2 booster.
